# Effects of homeostatic constraints on associative memory storage and synaptic connectivity of cortical circuits

**DOI:** 10.3389/fncom.2015.00074

**Published:** 2015-06-18

**Authors:** Julio Chapeton, Rohan Gala, Armen Stepanyants

**Affiliations:** Department of Physics and Center for Interdisciplinary Research on Complex Systems, Northeastern UniversityBoston, MA, USA

**Keywords:** perceptron, associative memory, *l*_0_ norm, *l*_1_ norm, inhibitory, critical capacity, synaptic weight, connection probability

## Abstract

The impact of learning and long-term memory storage on synaptic connectivity is not completely understood. In this study, we examine the effects of associative learning on synaptic connectivity in adult cortical circuits by hypothesizing that these circuits function in a steady-state, in which the memory capacity of a circuit is maximal and learning must be accompanied by forgetting. Steady-state circuits should be characterized by unique connectivity features. To uncover such features we developed a biologically constrained, exactly solvable model of associative memory storage. The model is applicable to networks of multiple excitatory and inhibitory neuron classes and can account for homeostatic constraints on the number and the overall weight of functional connections received by each neuron. The results show that in spite of a large number of neuron classes, functional connections between potentially connected cells are realized with less than 50% probability if the presynaptic cell is excitatory and generally a much greater probability if it is inhibitory. We also find that constraining the overall weight of presynaptic connections leads to Gaussian connection weight distributions that are truncated at zero. In contrast, constraining the total number of functional presynaptic connections leads to non-Gaussian distributions, in which weak connections are absent. These theoretical predictions are compared with a large dataset of published experimental studies reporting amplitudes of unitary postsynaptic potentials and probabilities of connections between various classes of excitatory and inhibitory neurons in the cerebellum, neocortex, and hippocampus.

## Introduction

**Figure 1:**
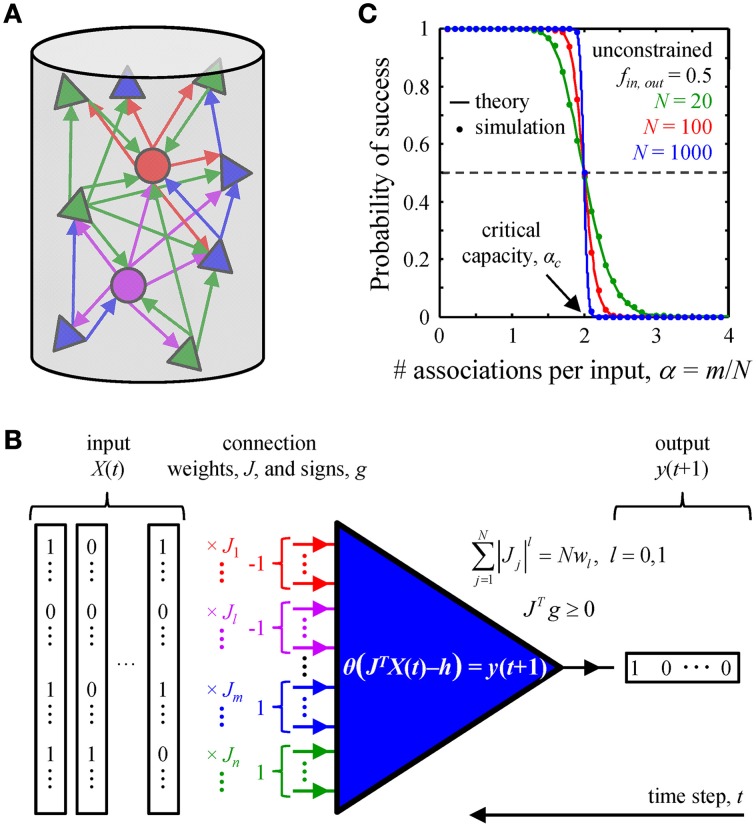
**Associative memory storage in local cortical circuits. (A)** A cortical column contains many classes of inhibitory (circles) and excitatory (triangles) neurons. **(B)** By adjusting the weights of their presynaptic connections, *J*, neurons in the column can learn to associate certain input patterns, *X*(*t*), with particular outputs, *y*(*t* + 1). Such changes in the connection weights are constrained by the homeostatic regulation of the overall weight, *Nw*_1_, or number, *Nw*_0_, of non-zero weight connections, as well as by the excitatory/inhibitory nature of individual presynaptic inputs, vector *g*. **(C)** A neuron's ability to learn a set of presented associations decreases with the number of associations in the set, *m*. The transition from perfect learning to inability to learn an entire set becomes very sharp with increasing number of potential inputs, *N*. Our numerical simulations (dots) are in good agreement with theoretical results (solid lines) (Cover, 1965). Critical capacity, α*_c_*, is defined as the number of associations per potential input, *m*/*N*, which can be learned with 0.5 probability of success. This capacity in the unconstrained perceptron model is 2.

It has long been known that learning and long-term memory formation in the brain are accompanied with changes in the patterns and weights of synaptic connections (see Bailey and Kandel, 1993; Chklovskii et al., 2004; Holtmaat and Svoboda, 2009 for review). Yet, a detailed understanding of the effects of learning on synaptic connectivity is still hindered by an insufficient account of network activity patterns and cell-type specific, experience-dependent learning rules. Thus, it is currently not feasible to directly relate the learning experience of an animal to specific changes in its synaptic connectivity. As an alternative, one may look for basic statistical features of synaptic connectivity which are catalyzed by the learning process, develop over time, and are present in adult circuits. In this study, we examine a biologically motivated, exactly solvable model of associative learning in an attempt to identify such connectivity features in local cortical circuits. Inspired by the ideas introduced by Gardner and Derrida (1988) and further developed by Brunel et al. (2004), we hypothesized that a given local circuit of the adult cortex is functioning in a steady-state. In this state the associative memory storage capacity of the circuit is maximal (critical) (Cover, 1965; Hopfield, 1982; Gardner, 1988; Gardner and Derrida, 1988), and learning new associations is accompanied with forgetting some of the old ones (Figure 1).

The steady-state learning hypothesis is supported by computational studies conducted in the cerebellar (Brunel et al., 2004; Barbour et al., 2007) and cerebral (Chapeton et al., 2012) cortices. It is also consistent with recent experimental evidence from human subjects, showing that new learning and memory retrieval can be accompanied by forgetting (Kuhl et al., 2010; Wimber et al., 2015). Thus, ongoing activity in the brain is likely to present neurons with sets of associations that are much larger than the learning capacity of the neurons. Consequently, the neurons will learn (presumably in development) as much as they possibly can and from then on (throughout adulthood) will remain at their critical memory storage capacity. This seemingly trivial hypothesis has very powerful implications. Because the properties of the network in the steady-state are independent of the learning path taken by the network to reach that state, one can analyze the steady-state connectivity in the absence of detailed knowledge of the animal's experience or the learning rules involved. This will remain true as long as the animal's experience is rich enough to present the network with a number of associations that exceeds the network's capacity, and the learning rules are versatile enough to learn the critical number of associations within the developmental period. Due to the fundamental nature of this hypothesis its predictions are expected to hold for many species, brain areas, and learning conditions.

In what follows, we extend the steady-state learning model described in Chapeton et al. (2012) by considering multiple classes of excitatory and inhibitory neurons and by incorporating biologically motivated homeostatic constraints. There is emerging evidence suggesting that in spite of circuit changes which accompany learning, individual cells may regulate (*i*) the total number (*l*_0_ norm) and/or (*ii*) the overall weight (*l*_1_ norm) of their presynaptic inputs. For example, it has been shown that the numbers of excitatory and inhibitory synapses onto excitatory cells in the adult cortex remain constant over periods of many days to weeks (Holtmaat et al., 2005, 2006; Fuhrmann et al., 2007; Brown et al., 2009; Hofer et al., 2009; Kim and Nabekura, 2011; Chen et al., 2012). Similarly, it has been shown that synapse loss can be counterbalanced by enlargement of other synapses, such that the summed synaptic surface area per length of dendritic segment remains constant across time and conditions (Bourne and Harris, 2011). In addition, long-term imaging studies have reported that total spine volume, as measured by normalized brightness, remains constant over days (Holtmaat et al., 2006; Kim and Nabekura, 2011). Because spine volume is correlated with synaptic weight (Matsuzaki et al., 2004; Arellano et al., 2007; Harvey and Svoboda, 2007; Zito et al., 2009), these findings suggest that the overall weight of presynaptic inputs remains constant throughout learning.

Below, we provide a detailed formulation of the homeostatically constrained steady-state learning model. The model was solved analytically and the solution was validated numerically. The results were compared with a large number of published experimental studies reporting probabilities of connections and distributions of connection weights for various classes of excitatory and inhibitory neurons in the cerebellum, neocortex, and hippocampus.

## Materials and methods

In this section we formulate a theoretical model of steady-state learning, which incorporates various classes of neurons and a number of biologically inspired constraints. Related models, which only include some of the constraints considered here, were previously described in a number of studies (e.g., Cover, 1965; Edwards and Anderson, 1975; Sherrington and Kirkpatrick, 1975; Gardner, 1988; Gardner and Derrida, 1988; Amit et al., 1989; Viswanathan, 1993; Brunel et al., 2004; Chapeton et al., 2012). Detailed description of theoretical and numerical methods can be found in Text S1.

### Biologically constrained model of steady-state learning

Networks in the cortex are thought to be organized in columnar units. Such units may include various functional (Hubel and Wiesel, 1963, 1977) and structural columns (Lübke and Feldmeyer, 2007; Stepanyants et al., 2008), which are typically a few hundred micrometers in radius. Analyses of neuron morphology (Kalisman et al., 2003; Binzegger et al., 2004; Stepanyants and Chklovskii, 2005; Stepanyants et al., 2008) have shown that the mesh created by the axonal and dendritic arbors of cells within such units contains numerous micron-size axo-dendritic appositions, which are called potential synapses. A pair of potentially connected cells can form a synaptic connection through local structural synaptic plasticity (Stepanyants et al., 2002; Trachtenberg et al., 2002; Escobar et al., 2008). Though nearby neurons (e.g., separated by less than 50 μm) within cortical units are typically interconnected in terms of potential synapses, functional synaptic connectivity is invariably sparse (Thomson and Lamy, 2007). For the purpose of this study we consider two cells to be functionally connected if an action potential fired by the presynaptic cell elicits a detectable response in the cell body of the postsynaptic neuron. Such a response, measured as a deviation of the membrane potential from its resting value, is referred to as a unitary postsynaptic potential (uPSP). The sign of a uPSP in a cortical neuron is dependent on the class of the presynaptic cell; it is positive if the presynaptic cell is excitatory (uEPSP) and negative if it is inhibitory (uIPSP).

We consider a local cortical network involved in an associative learning task (Figure 1A). The network may contain various excitatory and inhibitory neuron classes which are characterized by distinct firing probabilities. The state of the network at time *t*, *X*(*t*), is described by the binary (0 or 1) activities of all neurons. The network must learn to associate this state with the subsequent network state *X*(*t* + 1), and that to the state at the following time step, *X*(*t* + 2), etc., thus learning a chain of associated network states, *X*(*t*) → *X*(*t* + 1) → … *X*(*t* + *m*). Assuming that the successive network states are uncorrelated (see the next subsection) one can reduce the problem of network learning to the problem of learning by individual neurons (Figure 1B).

Thus, we consider a single model neuron, which receives *N* potential inputs from *N* potentially presynaptic partners and is faced with a task of learning a set of *m* input-output associations. The inputs, enumerated with index *j*, may come from various excitatory and inhibitory neuron classes which have characteristic firing probabilities, *f_j_*. The model neuron is motivated by the McCulloch and Pitts model (McCulloch and Pitts, 1943):

(1) $$\theta \left(\sum_{j=1}^{N}J_{j}X_{j}^{\mu }-h\right)=y^{\mu },\;\mu =1,\ldots ,m$$

Here, *J_j_* is the weight of presynaptic input *j*, *h* is the firing threshold of the neuron, and θ denotes the Heaviside function. The inputs, *X*^μ^_*j*_ (μ = 1, …, *m*), and outputs, *y^μ^*, are binary and their values are randomly drawn from neuron-class dependent probability distributions: 0 with probability 1 - *f_j_* and 1 with probability *f_j_*. The term $\sum_{j\;=\;1}^{N}{\left|J_{j}\right|}^{l}=Nw_{0}$ plays the role of the postsynaptic potential, and the neuron fires when this potential exceeds *h*. Equation (1) can be rewritten as a set of inequalities:

(2) $$\left(2y^{\mu }-1\right)\left(\sum_{j=1}^{N}J_{j}X_{j}^{\mu }-h\right)>0,\;\mu =1,\ldots ,m$$

In this study we impose the following biologically inspired constraints on the learning of associations $\left\{X_{j}^{\mu },y^{\mu }\right\}$:

(1) The weights of presynaptic inputs, *J_j_*, are sign-constrained in a way that is determined by the class of individual inputs,

(3) $$J_{j}g_{j}\geq 0,\;j=1,\ldots ,N$$

In these inequalities, *g_j_* = 1 if input *j* is excitatory and *g_j_* = −1 if it is inhibitory.

(2) The weights of input connections are also constrained to have a fixed norm. In the following we restrict the analysis to two cases: (*i*) *l*_0_ norm constraint, which corresponds to learning with a fixed number of non-zero weight inputs and is defined in the limit, $\underset{l\to 0}{\lim}\sum_{j\;=\;1}^{N}{\left|J_{j}\right|}^{l}=Nw_{0}$, and (*ii*) *l*_1_ norm constraint, which corresponds to learning with a fixed overall magnitude of the input weights, $\sum_{j\;=\;1}^{N}\left|J_{j}\right|=Nw_{1}$. In these expressions, *w*_0_ is referred to as the overall connection probability, while *w*_1_ is the average absolute connection weight. For conciseness, the *l*_0_ and *l*_1_ norm constraint can be combined into a single equation:

(4) $$\sum_{j=1}^{N}{\left|J_{j}\right|}^{l}=Nw_{l},\;l=0,1$$

(3) The firing threshold of the neuron, *h*, is fixed and does not change during learning.

(4) Associations, $\left\{X_{j}^{\mu },y^{\mu }\right\}$, must be learned robustly, which means that the postsynaptic potential must be somewhat above (below) the firing threshold if *y*^μ^ = 1 (0). This imposed minimal deviation from the threshold is referred to as the robustness parameter, κ ≥ 0. To incorporate this parameter, we modify the r.h.s of Equation (2), making the inequalities more stringent:

(5) $$\left(2y^{\mu }-1\right)\left(\sum_{j=1}^{N}J_{j}X_{j}^{\mu }-h\right)>\kappa ,\;\mu =1,\ldots ,m$$

To summarize, the full model can be reduced to the following:

(6) $$\begin{array}{l} \left(2y^{\mu }-1\right)\left(\sum_{j=1}^{N}J_{j}X_{j}^{\mu }-h\right)>\kappa ,\;\mu =1,\ldots ,m\\ J_{j}g_{j}\geq 0,\;j=1,\ldots ,N\\ \sum_{j=1}^{N}{\left|J_{j}\right|}^{l}=Nw_{l},\;l=0,1\\ \text{Prob}\left(X_{j}^{\mu }\right)=\{\begin{array}{ll} 1-f_{j}, & X_{j}^{\mu }=0\\ f_{j}, & X_{j}^{\mu }=1 \end{array}\\ \text{Prob}\left(y^{\mu }\right)=\{\begin{array}{ll} 1-f_{out}, & y^{\mu }=0\\ f_{out}, & y^{\mu }=1 \end{array} \end{array}$$

Any set of connection weights, *J_j_*, which satisfy Equation (6) is a valid solution of model.

### Model assumptions and approximations

The steady-state learning model relies on several assumptions and approximations. Here we describe these assumptions and provide experimental evidence supporting the approximations made:

(1) We discretized time into finite-size bins and describe the activity of neurons in the network with binary values: 1 if a neuron is firing and 0 if a neuron is silent. This approximation is reasonable so long as one can choose an integration window which is larger than the duration of a typical uPSP (τ), yet small enough not to encompass successive action potentials fired by any given cell. Denoting the typical firing rate for a cell class with *r*, such binning of activity should be possible when *r* × τ is smaller than one. In fact, many classes of neurons maintain *in vivo* firing rates that are low enough for this condition to be valid. Specifically, uEPSPs and uIPSPs in pyramidal cells typically have τ in the 40–60 ms range (Sayer et al., 1990; Markram et al., 1997; Gonzalez-Burgos et al., 2005; Sun et al., 2006; Lefort et al., 2009), while the spontaneous firing rates of these cells *in vivo* are *r* ~ 1–3 Hz (Csicsvari et al., 1999; Puig et al., 2003; Hromadka et al., 2008; Yazaki-Sugiyama et al., 2009, also see Barth and Poulet, 2012 for review). These observations put cortical pyramidal cells well within the range of validity of the above approximation. For inhibitory cells the data is more variable, but generally also supports the approximation. For example, the reported spontaneous firing rates *in vivo* are 9.2 Hz for FS cells in mouse visual cortex (Yazaki-Sugiyama et al., 2009), 7.6 Hz for FS cells in cat striate cortex (Azouz et al., 1997), about 3 Hz for PV cells and <1 Hz for SOM cells in mouse visual cortex (Ma et al., 2010), and 13–14 Hz in CA1 interneurons of rat hippocampus (Csicsvari et al., 1999). We note that it is not clear if the activity of neurons during associative learning resembles low firing rate spontaneous activity, or whether it is similar to the bursting activity of subsets of neurons recorded in animals actively engaged in trained behaviors. Nonetheless, because the fraction of bursting neurons at any given time is small (Barth and Poulet, 2012), the average network firing rate is expected to be low. For example, *in vivo* imaging studies, in which the activities of large ensembles of cortical neurons are monitored over time, have reported population average firing rates of <1 Hz (Kerr et al., 2005; Greenberg et al., 2008; Golshani et al., 2009).

(2) We used linear summation to approximate integration of uPSPs in the cell body. This has been shown to be a good approximation in the neocortex (Tamas et al., 2002; Leger et al., 2005; Araya et al., 2006), cerebellum (Brunel et al., 2004), and hippocampus (Cash and Yuste, 1998, 1999).

(3) We assumed that the threshold of each neuron remains fixed throughout learning. This assumption was motivated by the fact that coefficients of variation in the values of firing thresholds of cortical excitatory and inhibitory neurons are several-fold smaller than the corresponding numbers for the uPSP amplitudes. For example, coefficients of variation for the numerous cortical projections summarized in Supporting Tables 1 and 2 of Chapeton et al. (2012) have the following average values: 0.17 ± 0.02, (mean ± SE, *n* = 9 systems) for firing thresholds and 0.94 ± 0.03 (*n* = 52 systems) for connection weights.

(4) We followed Dale's principle (Dale, 1935) and assumed that the weights of excitatory/inhibitory inputs remain positive/negative throughout learning.

(5) The activities of all neurons in the network (*j* = 1, …, *N*) at every time step, μ, were randomly drawn from neuron-class specific probability distributions, Prob $\left(X_{j}^{\mu }\right)$, leading to successive network states that are (*i*) independent and (*ii*) random. With this approximation, the problem of learning by the network was decoupled and reduced to the problem of learning by *N* independent neurons. This approximation is supported by the following experimental observations. (*i*) For cortical neurons *in vivo*, serial correlation coefficients of inter-spike intervals are known to be small. For example, correlations of all lags greater than one are not significantly different from zero (Nawrot et al., 2007; Engel et al., 2008). Although small, but significant, lag one correlations (~ −0.2) are observed at high firing rates (Nawrot et al., 2007), these correlations vanish at <2 Hz (Engel et al., 2008). (*ii*) Correlations between the activities of pairs of cells *in vivo* are known to be small. For example, low pairwise correlations have been reported for pyramidal cells in rat olfactory (~0.05) (Miura et al., 2012) and visual (~0.033) (Greenberg et al., 2008) cortices. Weak pairwise correlations have also been found in the sensorimotor cortex of behaving monkeys and humans (Truccolo et al., 2010). In addition, extracellular recordings from L2/3 of somatosensory cortex have shown that correlation coefficients between regular spiking cells are small during periods of spontaneous and evoked activity (0.04 and 0.02) (Middleton et al., 2012). Similar results have been obtained for the correlations between regular spiking and fast spiking cells (0.11 and 0.01) (Middleton et al., 2012).

(6) The *l*_0_ and *l*_1_ norm constraints were motivated by the following experimental evidence. (*i*) The density of spines on excitatory neuron dendrites remains constant over days to weeks in many areas of the adult cortex (Holtmaat et al., 2005, 2006; Fuhrmann et al., 2007; Brown et al., 2009; Hofer et al., 2009; Kim and Nabekura, 2011). Likewise, the number of inhibitory synapses onto excitatory dendrites (Chen et al., 2012) and the number of spines on some inhibitory cell dendrites (Keck et al., 2011) remain nearly constant over days. Together, these studies suggest that homeostatic mechanisms may regulate the number of synapses received by excitatory and inhibitory neurons (*l*_0_ norm constraint). (*ii*) It has been reported that the total size of spines remains constant over several days as measured by the normalized spine brightness (Holtmaat et al., 2006; Kim and Nabekura, 2011). Because the normalized spine brightness is correlated with spine volume (Holtmaat et al., 2005) and the latter is correlated with synaptic weight (Matsuzaki et al., 2004; Arellano et al., 2007; Harvey and Svoboda, 2007; Zito et al., 2009), the overall weight of the presynaptic inputs of a pyramidal cell may be conserved. Another study (Bourne and Harris, 2011) has reported that by 2 h after LTP induction dendrites of CA1 pyramidal neurons in the hippocampus lose some of their small dendritic spines. However, this loss is balanced by an enlargement of the surface area of other excitatory synapses in such a way that the summed surface area of excitatory synapses remained constant across time and conditions. A similar trend was observed for the inhibitory synapses (Bourne and Harris, 2011). These observations imply that dendrites may use local protein synthesis to maintain the overall weight of excitatory and inhibitory inputs (*l*_1_ norm constraint).

(7) We assumed that associative memories can be recalled robustly in the presence of small noise in synaptic transmission, e.g., failures in generation or propagation of presynaptic action potentials, spontaneous neural activity, synaptic failure, and fluctuations in synaptic weight. In order to incorporate this feature into the model we assumed that an association was robustly learned by a neuron if it could be correctly recalled even in the presence of fluctuations in postsynaptic potential of size κ.

### Theoretical solution of the model

The theoretical solution of the model, Equation (6), is governed by four variables (*u*_+_, *u*_−_, *z*, and *x*), which are implicitly defined by the following system of equations:

(7) $$\begin{array}{l} f_{out}I_{1}\left(-u_{-},0\right)=(1-f_{out})I_{1}\left(-u_{+},0\right)\\ \frac{1}{N}\sum_{i=1}^{N}{\left(\frac{1}{\sqrt{f_{i}\left(1-f_{i}\right)}}\right)}^{l}I_{l}(g_{i}z\sqrt{\frac{f_{i}}{1-f_{i}}}+\frac{\left(\sqrt{N}\kappa x-hz\right)}{Nw_{1}\sqrt{f_{i}\left(1-f_{i}\right)}}\delta_{l,1},\\ \;\;\sqrt{\frac{2\left(\sqrt{N}\kappa x-hz\right)Q}{hw_{0}}}\delta_{l,0})={\left(\frac{N}{h}\right)}^{l}w_{l}Q^{l}\\ \frac{1}{N}\sum_{i=1}^{N}\frac{f_{i}g_{i}}{\sqrt{f_{i}\left(1-f_{i}\right)}}I_{1}(g_{i}z\sqrt{\frac{f_{i}}{1-f_{i}}}+\frac{\left(\sqrt{N}\kappa x-hz\right)}{Nw_{1}\sqrt{f_{i}\left(1-f_{i}\right)}}\delta_{l,1},\\ \;\sqrt{\frac{2\left(\sqrt{N}\kappa x-hz\right)Q}{hw_{0}}}\delta_{l,0})=Q\\ \frac{1}{N}\sum_{i=1}^{N}I_{2}(g_{i}z\sqrt{\frac{f_{i}}{1-f_{i}}}+\frac{\left(\sqrt{N}\kappa x-hz\right)}{Nw_{1}\sqrt{f_{i}\left(1-f_{i}\right)}}\delta_{l,1},\\ \;\;\sqrt{\frac{2\left(\sqrt{N}\kappa x-hz\right)Q}{hw_{0}}}\delta_{l,0})=\frac{2Q^{2}N\kappa^{2}}{{\left(u_{+}+u_{-}\right)}^{2}h^{2}}\\ Q=2h\frac{\left(u_{+}+u_{-}\right)}{\sqrt{N}\kappa }\frac{f_{out}I_{0}\left(-u_{-},0\right)+(1-f_{out})I_{0}\left(-u_{+},0\right)}{f_{out}I_{1}\left(-u_{-},0\right)+(1-f_{out})I_{1}\left(-u_{+},0\right)}x\\ u_{+}+u_{-}\geq 0;\;x\geq 0;\;\left(\sqrt{N}\kappa x-hz\right)\delta_{l,0}\geq 0 \end{array}$$

Detailed derivation of these equations, together with the definitions of the special functions *I*_0, 1, 2_, can be found in Text S1.

The critical capacity of a neuron, the probabilities of its non-zero weight connections for different input classes (denoted with *i*), *P^con^_i_*, and the probability density functions for its non-zero input weights, *p_i_* (*J*), can be expressed in terms of these four variables:

(8) $$\begin{array}{l} \;\alpha_{c}=2x^{2}\frac{\left(f_{out}I_{2}\left(-u_{-},0\right)+(1-f_{out})I_{2}\left(-u_{+},0\right)\right)}{{\left(f_{out}I_{1}\left(-u_{-},0\right)+(1-f_{out})I_{1}\left(-u_{+},0\right)\right)}^{2}}\\ \;P_{i}^{con}=I_{0}\left(g_{i}z\sqrt{\frac{f_{i}}{1-f_{i}}}+\frac{\left(\sqrt{N}\kappa x-hz\right)}{Nw_{1}\sqrt{f_{i}\left(1-f_{i}\right)}}\delta_{l,1}+\sqrt{\frac{2\left(\sqrt{N}\kappa x-hz\right)Q}{hw_{0}}}\delta_{l,0},0\right)\\ p_{i}\left(J\right)=\frac{\sqrt{f_{i}\left(1-f_{i}\right)}}{2\sqrt{\pi }I_{0}\left(g_{i}z\sqrt{\frac{f_{i}}{1-f_{i}}}+\frac{\left(\sqrt{N}\kappa x-hz\right)}{Nw_{1}\sqrt{f_{i}\left(1-f_{i}\right)}}\delta_{l,1}+\sqrt{\frac{2\left(\sqrt{N}\kappa x-hz\right)Q}{hw_{0}}}\delta_{l,0},0\right)}\\ \;\times {\left(Q-\frac{2\left(\sqrt{N}\kappa x-hz\right)}{f_{i}\left(1-f_{i}\right)w_{0}}\frac{h\delta_{l,0}}{N^{2}J^{2}}\right)}_{+}\\ \;\times \;e^{-{\left(\frac{\sqrt{f_{i}\left(1-f_{i}\right)}Q}{2}\frac{N}{h}J+z\sqrt{\frac{f_{i}}{1-f_{i}}}+\frac{\left(\sqrt{N}\kappa x-hz\right)}{h\sqrt{f_{i}\left(1-f_{i}\right)}}\left(\frac{hg_{i}\delta_{l,1}}{Nw_{1}}+\frac{h\delta_{l,0}}{w_{0}NJ}\right)\right)}^{2}} \end{array}$$

**Figure 2:**
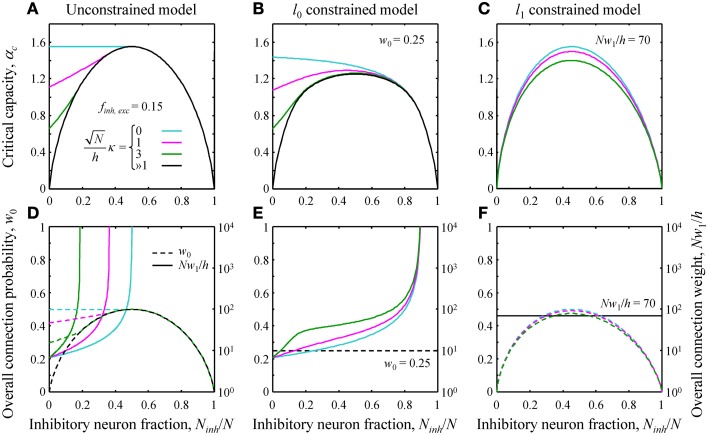
**(A–C)** Critical capacity as a function of the fraction of potential inhibitory inputs for *f_inh_* = *f_exc_* = 0.15 and various values of the robustness parameter κ. **(A)** The unconstrained model. At certain values of *N_inh_*/*N*, the curves merge with the asymptotic solution (black curve) corresponding to the limit of large robustness, $\sqrt{N}\kappa /h$≫1. For smaller fractions of inhibitory inputs, the critical capacity is a decreasing function of κ and an increasing function of *N_inh_*/*N*. **(B)** Qualitatively similar results were obtained in the *l*_0_ constrained model. **(C)** In the *l*_1_ constrained model the critical capacity curves are slightly skewed to the left and have a maximum at *N_inh_*/*N* < 0.5 for all values of κ. Values of the constraints are *w*_0_ = 0.25 in B and *Nw*_1_/*h* = 70 in **(C)**. **(D–F)** Overall connection probability (dashed lines) and overall connection weight (solid lines) as a function of *N_inh_*/*N*. Note the different *y*-axis scales for *w*_0_ (linear, left) and *Nw*_1_/*h* (logarithmic, right). In the constrained models, the overall connection probability **(E)** or the overall connection weight **(F)** is fixed for all values of *N_inh_*/*N* (horizontal lines).

**Figure 3:**
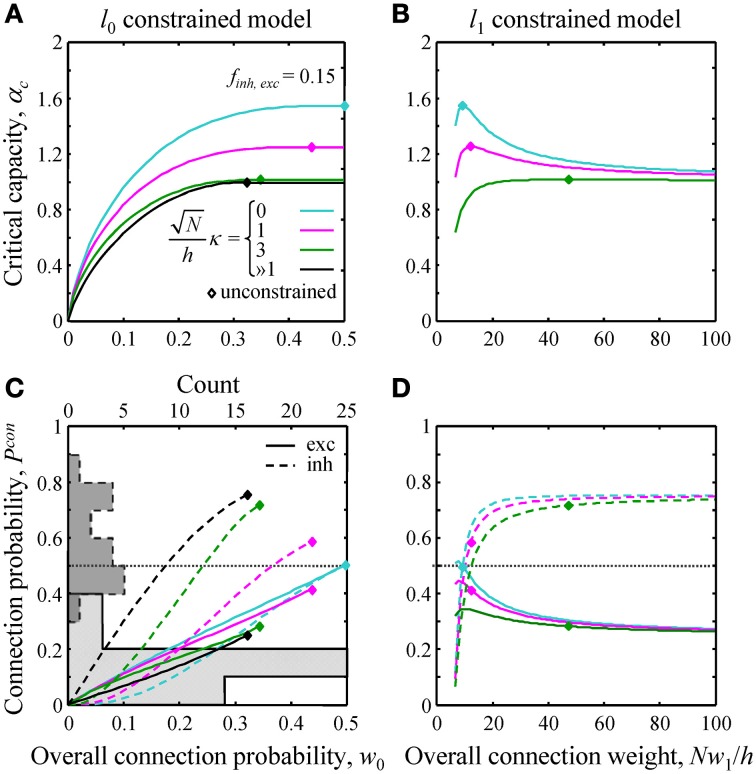
**Effects of** ***l*****_0_ and** ***l*****_1_ constraints on critical capacity and connection probability. (A,B)** Critical capacity of the *l*_0_ and *l*_1_ constrained models plotted as a function of the constraints, *w*_0_ and *Nw*_1_/*h*. Diamonds denote the corresponding value of the critical capacity in the unconstrained model. The critical capacity is at its maximum when *w*_0_ and *w*_1_ match the corresponding values calculated for the unconstrained model. **(C,D)** Excitatory (solid lines) and inhibitory (dashed lines) connection probabilities in the *l*_0_ and *l*_1_ constrained models. Gray histograms in **(C)** represent excitatory (solid outline) and inhibitory (dashed outline) connection probability data from a large set of experimental studies (see Text S1 for details). Note that the histogram counts are shown at the top of **(C)**.

Plus-sign in the subscript of the last equation denotes the positive part function. Corresponding results for the unconstrained case are included in Text S1. Equations (7) and (8) were solved with custom MatLab code (Theoretical_Results.m of Supplementary Materials) to produce the results shown in Figures 2, 3, and Figure S1.

### Model parameters

Results of the model, Equation (8), depend on the following dimensionless parameters: fraction of potential inputs of each class, *N_i_*/*N*, firing probabilities of these input classes, *f_i_*, robustness of the postsynaptic neuron, $\sqrt{N}\kappa /h$, and the values of norm constraints, *w*_0_ and *Nw*_1_/*h*. In Results, we only consider two classes of inputs, inhibitory and excitatory, and thus, the number of independent parameters in the unconstrained model reduces to four (*N_inh_*/*N*, *f_inh_*, *f_exc_*, $\sqrt{N}\kappa /h$). An additional parameter, *w*_0_ (*Nw*_1_/*h*), is present in the *l*_0_ (*l*_1_) norm constrained case.

**Figure 4:**
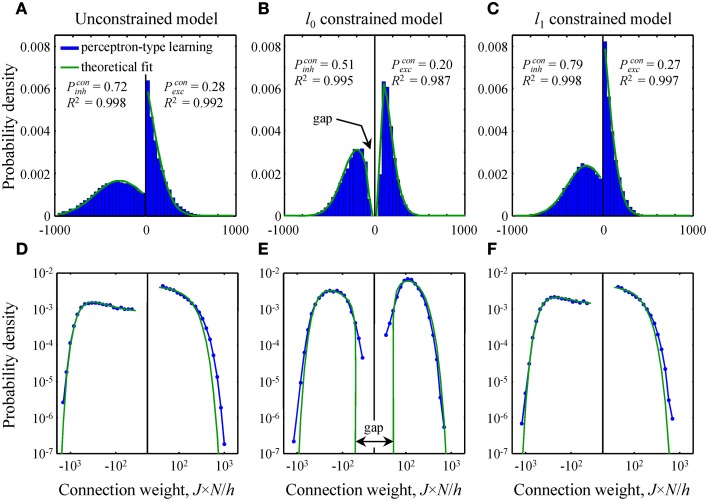
**Comparison of theoretical distributions of connection weights to numerical simulations. (A–C)** Probability density functions obtained with perceptron-type learning rules for *N* = 500, *N_inh_*/*N* = 0.15, $\sqrt{N}\kappa /h$ = 3, and f*_inh, exc_* = 0.15 are shown with blue bars. Values of the constraints are *w*_0_ = 0.25 in B and *Nw*_1_/*h* = 70 in C. Green lines show theoretical fits of these probability density functions with Equation (10). Goodness of the fits is captured by the high adjusted *R*^2^ coefficients. The theoretical distributions of excitatory and inhibitory connection weights in the unconstrained **(A)** and *l*_1_ constrained **(C)** models consist of Gaussians truncated at *J* = 0 and finite fractions of zero-weight connections. The distribution in the *l*_0_ constrained model **(B)** also contains a finite fraction of zero-weight connections, but is non-Gaussian. This distribution has gaps between zero and non-zero connection weights for excitatory and inhibitory inputs. Parameters *P^con^_inh,exc_* give theoretical fractions of inhibitory and excitatory non-zero weight connections. **(D–F)** Same probability density functions plotted on a log-log scale show deviations between theory and numerical simulations in the head and tail regions of the distributions.

The fraction of potential inhibitory inputs received by a neuron in the network, *N_inh_*/*N*, can be approximated by the average fraction of inhibitory neurons in the cortical column. The latter is known to be in the 0.11–0.20 range (Braitenberg and Schüz, 1998; Lefort et al., 2009; Meyer et al., 2011; Sahara et al., 2012). Thus, we used *N_inh_*/*N* = 0.15 in Figures 3, 4. Firing probabilities can be estimated based on the expression *f* = *r* × τ. Numerical values of firing rates, *r*, and integration windows, τ, for excitatory and inhibitory neurons are given in point 1 of Model Assumptions and Approximations subsection. Based on these numbers we estimated that *f_exc_* ≈ 0.1, while *f_inh_* is expected to be larger due to generally higher firing rates of inhibitory neuron classes. However, because the exact values of firing probabilities are not known, in Results we decided to adopt *f_inh_* = *f_exc_* = 0.15 (Figures 2–4), while in Supplementary Materials we show the results for the unbiased case, *f_inh_* = *f_exc_* = 0.5 (Figure S1). We did not find a clear way to determine the value of robustness parameter, $\sqrt{N}\kappa /h$, from experimental data. This is why, in Figures 2, 3 we first show that the results of the model depend on the value of this parameter in a predictable way, and then set $\sqrt{N}\kappa /h$= 3 in Figure 4.

The biologically plausible ranges for the dimensionless constraints, *w*_0_ and *Nw*_1_/*h*, were approximated based on their definitions (see Text S1). For two classes of presynaptic inputs these definitions yield:

(9) $$\begin{array}{l} w_{0}=\frac{N_{inh}}{N}P_{inh}^{con}+\left(1-\frac{N_{inh}}{N}\right)P_{exc}^{con}\\ \frac{Nw_{1}}{h}=\frac{N}{h}\left(\frac{N_{inh}}{N}P_{inh}^{con}\langle J_{inh}\rangle +\left(1-\frac{N_{inh}}{N}\right)P_{exc}^{con}\langle J_{exc}\rangle \right) \end{array}$$

**Figure 5:**
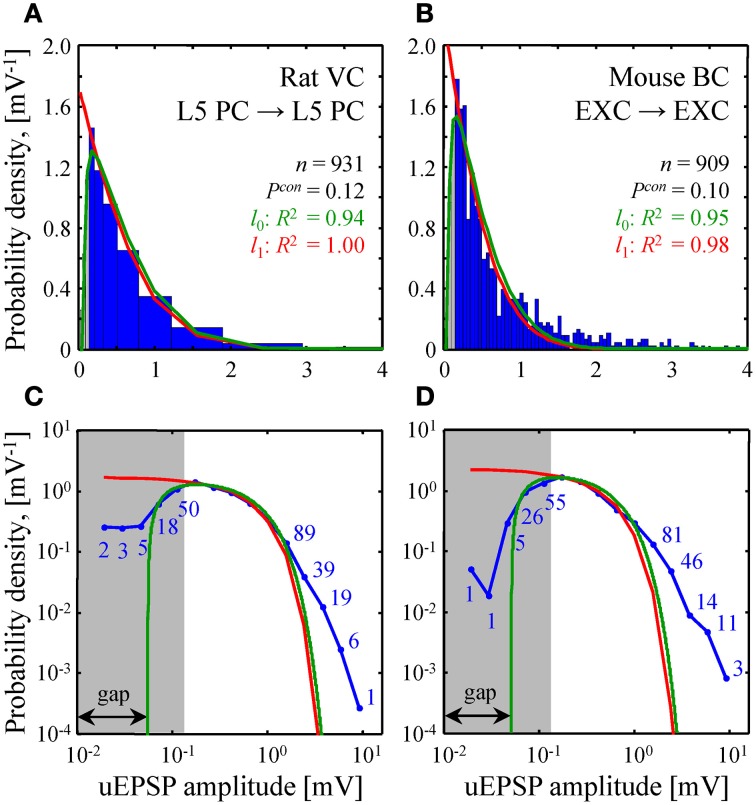
**Comparison of theoretical and experimental distributions of connection weights. (A,B)** Blue bars show distributions of uEPSP amplitudes in layer 5 of rat visual cortex (Song et al., 2005) and all layers of mouse barrel cortex (Lefort et al., 2009). These distributions were fitted with both the *l*_0_ (green) and *l*_1_ (red) constrained models (see Text S1 for details). Goodness of the fits is captured by the adjusted *R*^2^ coefficients, which are close to 1. **(C,D)** To examine how the two models fit the heads and the tails of the distributions of uEPSP amplitudes (blue line), **(A,B)** are re-plotted on a log-log scale. Blue numbers indicate the counts in the logarithmic bins. Data points, which fall below the uEPSP detection threshold and are thus unreliable, are highlighted in gray. The probability density in the *l*_0_ model has a gap between zero and non-zero connection weights; there is no such gap in the *l*_1_ model.

Here *P^con^_inh,exc_* and $\langle J_{inh,exc}\rangle$ are the connection probabilities and the average uPSP amplitudes of inhibitory and excitatory inputs. To estimate the values of the constraints we combined the dataset compiled in Chapeton et al. (2012) with a recent study of inhibitory connectivity (Packer and Yuste, 2011) and then restricted the analysis to neocortical systems. The 95% confidence intervals were then obtained using bootstrap sampling with replacement (*n* = 10,000 samples). Parameters *h*, *P^con^_inh,exc_*, and $\langle J_{inh,exc}\rangle$ were sampled with weights proportional to the numbers of experimental counts, whereas *N* and *N_inh_*/*N* were sampled uniformly from 5,000 to 10,000 (Lefort et al., 2009; Meyer et al., 2010) and 0.11–0.20 (Braitenberg and Schüz, 1998; Lefort et al., 2009; Meyer et al., 2011; Sahara et al., 2012) intervals. This procedure resulted in 95% confidence intervals of [0.1–0.4] for *w*_0_ and [20–190] for *Nw*_1_/*h*. In Figure 3 we show how results of the model depend on the values of these constraints, while in Figures 2, 5 we opted to use the average values obtained from the bootstrap sampling, *w*_0_ = 0.25 and *Nw*_1_/*h* = 70.

### Numerical solutions of the model

Since the analytical calculations used to produce the results of this study are very involved we used numerical simulations as an additional validation step. Details of these numerical algorithms can be found in Text S1. Standard convex optimization methods were used to produce numerical solutions for the unconstrained and *l*_1_ norm constrained problems. Since the *l*_0_ norm constrained problem is non-convex, numerical solutions in this case were performed with a modified perceptron learning rule. The critical capacity (Figures S1A–C) and the distributions of connection weights (Figures S1D–F) resulting from these simulations are in good agreement with the theoretical calculations.

Numerical simulations were also used to illustrate plausibility of the steady-state learning hypothesis, which relies on the assumption that a network is able to reach the state of maximum memory storage capacity. To this end, we used perceptron-type learning rules and biologically plausible model parameters to reproduce theoretical results of all three cases (Figure 4).

### Fitting distributions of connection weights

Theoretical probability density functions of Equation (8) were used to fit simulated and experimental distributions of connection weights. To this end, these equations were rewritten by using the experimentally determined values of *P^con^_i_* and introducing two parameters: σ*_i_*, which describes the width of the distribution and *G_i_*, which is the magnitude of the minimum non-zero connection weight present in the *l*_0_ model. The resulting probability density functions are governed by two parameters (σ*_i_* and *G_i_*) in the *l*_0_ case and one parameter (σ*_i_*) in the *l*_1_ case:

(10) $$\begin{array}{l} \sigma_{i}=\frac{\sqrt{2}h}{\sqrt{f_{i}\left(1-f_{i}\right)}QN};\;G_{i}=\sqrt{\frac{2\left(\sqrt{N}\kappa x-hz\right)}{f_{i}\left(1-f_{i}\right)w_{0}Q}\frac{h}{N^{2}}}\\ p_{i}\left(J\right)=\frac{1}{\sqrt{2\pi }P_{i}^{con}}\frac{h}{N\sigma_{i}}{\left(1-\frac{G_{i}^{2}}{J^{2}}\delta_{l,0}\right)}_{+}\times \\ e^{-{\left(\frac{g_{i}J}{\sqrt{2}\sigma_{i}}+\text{erfinv}\left(1-2P_{i}^{con}\right)+\frac{\sqrt{2}G_{i}}{\sigma_{i}}\left(\frac{G_{i}}{2g_{i}J}-1\right)\delta_{l,0}\right)}^{2}} \end{array}$$

Fitting the simulated distributions of inhibitory/excitatory connection weights shown in Figure 4 was done with one parameter (σ*_inh_*/σ*_exc_*) in the unconstrained and *l*_1_ constrained models, and two parameters (σ*_inh_*/σ*_exc_* and *G_inh_*/*G_exc_*) in the *l*_0_ case. Fitting was done in MatLab by using non-linear least squares fit. We note that the functional form of the distribution of connection weights in the unconstrained model (Chapeton et al., 2012), written in the notation of Equation (10), is identical to that of the *l*_1_ case. For this reason, the unconstrained and *l*_1_ models produce identical fits.

A similar fit of experimental distributions of uEPSP amplitudes is shown in Figure 5. Fitting with the *l*_0_ model produced the following best fit parameters: σ = 1.06 [0.88–1.23] mV (mean [95% confidence interval]), *G* = 0.055 [0.045–0.064] mV in Figure 5A and σ = 0.79 [0.71–0.88] mV, *G* = 0.051 [0.041–0.060] mV in Figure 5B. For the *l*_1_ model we discarded weak, unreliable connections (gray regions in Figure 5) and thus introduced a normalization factor *A* as an additional fitting parameter. Hence, fitting in this case was also performed with two parameters. The resulting best fit values of σ were: 1.02 [0.92–1.13] mV in Figure 5A and 0.81 [0.70–0.92] mV in Figure 5B.

## Results

### Effects of homeostatic constraints on network capacity and connectivity

The general solution of the model is described in Text S1. Since this solution is very involved, theoretical results were validated with numerical simulations (see Figure S1). Here we illustrate the theoretical results by considering a single cell receiving inputs from two classes of presynaptic neurons, one inhibitory and one excitatory. The critical associative memory storage capacity of this cell, α_*c*_, was calculated by solving the system of Equations (7) and (8) (Theoretical_Results.m of Supplementary Materials). Figures 2A–C show the dependence of α_*c*_ in the unconstrained and *l*_0, 1_ norm constrained models on the fraction of potential inhibitory inputs, *N_inh_*/*N*, and the robustness parameter, κ. Though the results for the three models are distinctly different, there are notable common trends. First, in all three models, the critical capacity is a decreasing function of κ, indicating the trade-off between the maximum number of associations a neuron can learn and the robustness of the learned associations. Second, in the case of robust memory storage (κ > 0), adding a small fraction of inhibitory neurons increases α_*c*_. This, however, comes at the expense of the total number of non-zero weight connections, *Nw*_0_, and/or the overall connection weight, *Nw*_1_ (Figures 2D–F).

Next, we evaluated the effects of the *l*_0, 1_ norm constraints on the critical capacity and connection probabilities for various input classes. Figure 3 shows the results for two classes of inputs, excitatory and inhibitory, with *N_inh_*/*N* = 0.15. This numerical value, as well as the values of other parameters of the theory, is based on published experimental data (see Materials and Methods for details). As expected, the critical capacity of the constrained models is maximal when *w*_0_ (Figure 3A) and *w*_1_ (Figure 3B) match the corresponding values of these parameters in the unconstrained case (diamonds in the figure). This is because at these exact values of *w*_0_ and *w*_1_ the norm constraints are effectively removed, and the solutions of the constrained models reduce to that of the unconstrained case, which naturally has the maximum critical capacity.

Increasing *w*_0_ beyond this point in the *l*_0_ model has no effect on critical capacity (Figure 3A) because it is always possible to start with the connectivity of the unconstrained network, and then add a small number of infinitesimally weak connections which will have no effect on the learned associations, but will increase the overall connection probability to the desired value. As the capacity of the *l*_0_ model cannot be greater than the capacity of the unconstrained model, solutions constructed in this way have the maximum possible capacity, and thus are valid for large values of *w*_0_ (to the right of the diamonds in Figure 3A). However, because multiple solutions of this type exist, excitatory and inhibitory connection probabilities cannot be defined uniquely.

Numerous experimental studies have shown that the probabilities of local excitatory and inhibitory connections onto the principal cortical neurons (pyramidal and spiny stellate cells in the cerebral cortex and Purkinje cells in the cerebellum) are distinctly different. In particular, excitatory connections are sparse, with connection probabilities well below 0.5, while the inhibitory connection probabilities are generally much higher. This difference can be seen in the histograms of Figure 3C which summarize connection probabilities compiled in Chapeton et al. (2012) together with the data from a large study of parvalbumin positive to pyramidal cell connectivity (Packer and Yuste, 2011). Consistent with these observations, the probabilities of excitatory and inhibitory connections in the unconstrained model have been shown to be distinctly different (Chapeton et al., 2012), *P^con^_exc_* < 0.5 and *P^con^_inh_* > 0.5 (diamonds in Figures 3C,D). Therefore, we decided to examine if the constrained models considered in this study produce a similar trend. Figure 3C shows *P^con^_exc_* and *P^con^_inh_* in the *l*_0_ norm constrained model plotted as functions of the overall connection probability, *w*_0_. Both *P^con^_exc_* and *P^con^_inh_* increase with *w*_0_, however, *P^con^_exc_* always remains below 0.5, while *P^con^_inh_* exceeds 0.5 beyond certain values of *w*_0_. The range of *w*_0_ values estimated for excitatory cells in the neocortex is 0.1–0.4 (see Materials and Methods). In this range *P^con^_exc_* < 0.5, while *P^con^_inh_* is higher than *P^con^_exc_* in the case of robust memory storage (κ > 1). Connection probabilities in the *l*_1_ norm constrained model depend on the value of *Nw*_1_/*h* (Figure 3D). This parameter, estimated from the experimental data, is in the 20–190 range (see Materials and Methods). In this range *P^con^_exc_* < 0.5 and *P^con^_inh_* > 0.5 for all values of robustness. Thus, for biologically realistic values of *w*_0_ and *Nw*_1_/*h*, the connection probabilities produced by the homeostatically constrained models are consistent with the experimentally observed difference in probabilities of excitatory and inhibitory connections onto principal cells.

### Distribution of connection weights

In this subsection we compare and contrast the probability densities of input connection weights at critical capacity for the unconstrained and *l*_0, 1_ norm constrained models [see Text S1 and Equations (8) and (10)]. In the unconstrained and *l*_1_ norm constrained cases these probability densities consist of Gaussian functions, truncated at zero, and finite fractions of zero-weight connections (Figures 4A,C). The distribution of connection weights in the *l*_0_ norm constrained model also contains a finite fraction of zero-weight connections. However, the probability density function for non-zero connection weights is non-Gaussian (Figure 4B). Interestingly, this function has a gap for weak input connections, i.e., it does not contain non-zero connection weights below a certain threshold. We would like to point out that this feature of connection weights constrained by the *l*_0_ norm was previously reported by Bouten et al. (1990), who considered associative learning by a neuron receiving a single class of sign-unconstrained inputs, i.e., the inputs were not constrained to be excitatory or inhibitory.

Since the steady-state learning hypothesis relies on the assumption that the network can achieve the state of maximum associative memory storage capacity, we set out to show that this can be done with a biologically plausible learning rule. To this end, we attempted to reproduce the theoretical critical capacities and the shapes of theoretical connection weight distributions in the three models by using modified perceptron learning rules (see Materials and Methods). The simulations were performed for biologically plausible values of model parameters (see Materials and Methods) and about 95% of theoretical, maximum memory storage capacity was reached in all three models: α_*c*_ = 0.95/0.99 (numerical/theoretical) in the unconstrained model, 0.94/0.99 in the *l*_0_ model, and 0.97/1.01 in the *l*_1_ model.

The overall shape of numerical distributions generated at theoretical critical capacity (Figures 4A–C) was in good agreement with the theory, Equation (10). However, small deviations between theory and numerical simulations were observed in the head and tail regions of these distributions. To examine these deviations in more detail we re-plotted the distributions of connection weights on a log-log scale (Figures 4D–F). In the unconstrained and *l*_1_ norm constrained models the tails of the numerical distribution appear to be slightly heavier than theoretically predicted Gaussian tails (Figures 4D,F), while in the *l*_0_ case (Figure 4E) there is a slight deviation in the regions of weak inhibitory and excitatory connections. It is likely that this deviation results from the fact that the numerical simulations were performed for a large, yet finite number of potential inputs, *N* = 500, while theoretical distributions were obtained in the limit of infinite *N*.

### Comparison of experimental and theoretical connection weight distributions

The two homeostatically constrained models produce distinctly different distributions of connection weights. Below, we investigate how these distributions compare with the distributions of uPSP amplitudes reported in experimental studies. For this purpose, we selected two studies with very high counts of recorded uEPSPs, one performed in rat visual cortex (*n* = 931, layer 5) (Song et al., 2005) and the other in mouse barrel cortex (*n* = 909, all layers) (Lefort et al., 2009). The two models (green and red lines in Figures 5A,B) were used to fit both experimental distributions (blue histograms in Figures 5A,B). In spite of the fact that the goodness of these fits was high as measured by the adjusted *R*^2^ coefficients, significant deviations were observed between the distributions (*P* < 10^−12^ for both models in Figures 5A,B; Kolmogorov-Smirnov test), most noticeably in the head and tail regions. To focus on these differences, Figures 5C,D show the distributions on a log-log scale.

Due to the fluctuations in electrophysiological recordings, very weak connections between neurons cannot be detected reliably. Such connections are often missed or ignored, leading to a systematic underestimate of weak connection counts. The gray regions in Figure 5 highlight the values of uEPSP amplitudes which fall below the reliable uEPSP detection threshold [0.1–0.25 mV in rodent neocortex (Mason et al., 1991; Markram et al., 1997; Feldmeyer et al., 1999; Berger et al., 2009)]. Unfortunately, the difference between the distributions produced by the two models only becomes apparent inside the gray regions, and thus, it cannot be directly tested. In these regions of small uEPSP amplitudes the *l*_0_ model provides seemingly better fits to the experimental distributions, but the statistical significance of these results could not be verified based on the available data.

Both models of steady-state learning underestimate the counts of strong synaptic connections (uEPSP amplitudes > 1 mV). Though not very numerous (blue numbers in Figures 5C,D), these strong connections appear to be a characteristic feature of cortical connectivity. Sub-criticality of neural networks in the brain, non-linearity in the summation of presynaptic inputs (Brunel et al., 2004), as well as the effects of the finite size of local cortical networks (Chapeton et al., 2012) (e.g., Figure 4D, right) have been proposed as possible explanations for the discrepancy between the Gaussian tails of the theoretical probability density functions and the much heavier distribution tails observed experimentally. At present, we are unable to differentiate between these explanations.

## Discussion

The study of associative learning by artificial neural networks has a long history dating back to the work of McCulloch and Pitts who introduced one of the first binary neuron models (McCulloch and Pitts, 1943). Rosenblatt later showed that such a binary neuron, now termed the perceptron, can solve classification problems by learning to associate certain input patterns with specific outputs (Rosenblatt, 1957, 1958). The memory storage capacity of a simple perceptron (no constraints, *h* = 0 or learnable threshold, κ = 0) was calculated by Cover (1965), who used geometrical arguments to show that a simple perceptron can learn to associate 2*N* unbiased input-output patterns (*f_in_* = *f_out_* = 0.5). It was Hopfield who recognized that stable states in recurrent networks of binary neurons can be used as a mechanism for memory storage and recall (Hopfield, 1982). Subsequently, a general framework for the analysis of memory storage capacity was established by Gardner (1988), Gardner and Derrida (1988) who used the replica theory, originally developed for spin glass applications (Edwards and Anderson, 1975; Sherrington and Kirkpatrick, 1975), to solve the problem of robust learning (κ > 0) of arbitrarily biased associations. To model granule to Purkinje cell connectivity in the cerebellum, Brunel and colleagues constrained Gardner's solution by fixing the firing threshold and forcing the inputs to be all excitatory (*J_j_* ≥ 0) (Brunel et al., 2004). These results were then extended by Chapeton et al. (2012) on the case of excitatory and inhibitory inputs and applied to cortical circuits.

In this study, we generalize the model of Chapeton et al. (2012) by incorporating multiple classes of excitatory and inhibitory neurons and including two types of experimentally motivated homeostatic constraints. The constraints were designed to ensure that individual neurons maintain a fixed total number or a fixed overall weight of their non-zero inputs throughout learning. Both constrained models were solved analytically by using the replica theory. The results were validated with numerical simulations and compared to the available data on cortical connectivity.

Our theory produced two specific results regarding the connectivity among potentially connected cells in steady-state networks. First, we showed that functional excitatory connections onto principal cells should be realized with less than 50% probability, while the probabilities of inhibitory connections should be higher (Figures 3C,D). Because in cortical systems inhibitory cells account for only 11–20% of all neurons, functional connectivity in a steady-state network is expected to be sparse, i.e., it must contain a large fraction of zero-weight connections. This theoretical finding is in qualitative agreement with a dataset compiled from 38 published studies (62 projections in total) in which connection probabilities have been measured in various cortical systems (histograms in Figure 3C). It is important to note that a zero-weight connection between neurons does not necessarily imply that the structural connection is absent, as the neurons may still be connected with synapses that are silent (synapses devoid of AMPA receptors Malinow and Malenka, 2002). This detail, however, did not factor into the comparison because neither the theory nor the electrophysiological recordings discriminate between the two alternatives. Furthermore, it has been shown that the fraction of silent synapses in adult cortex is low (e.g., Busetto et al., 2008).

Second, we derived the shapes of the connection weight distributions in a steady-state [see Equation (10) and Figures 4, 5]. Similar to the unconstrained model, distribution in the *l*_1_ case consists of Gaussians truncated at zero and a finite fraction of zero-weight connections. The distribution in the *l*_0_ model also contains a finite fraction of zero-weight connections, but the shape of the distribution for non-zero connection weights is no longer Gaussian. It is characterized by a gap between zero and non-zero connection weights. Hence, we predict that a network operating in a steady-state, subject to a constraint on the number of functional connections cannot contain arbitrarily small connection weights. Rather, there should be no functional connections weaker than a certain threshold (~0.05 mV for uEPSP amplitudes in the neocortex, Figure 5). It is not impossible to envision a biological mechanism by which weak connections can be silenced and/or completely eliminated (Oh et al., 2013). In practice, however, it may be difficult to test this hypothesis because the connection weight threshold is too small to be measured reliably with current experimental techniques (Mason et al., 1991; Markram et al., 1997; Feldmeyer et al., 1999; Berger et al., 2009). Interestingly, the value of the connection weight threshold obtained in this study is in agreement with the smallest quantal sizes and miniature EPSP amplitudes recorded in cortical pyramidal neurons [~0.1 mV (Hardingham et al., 2010)], responses believed to be produced by the release of neurotransmitter from a single presynaptic vesicle.

On the whole, the shapes of model distributions for non-zero connection weights are consistent with the experimental distributions of uEPSP amplitudes in rodent neocortex (Figure 5). However, significant discrepancies were observed in the head and tail regions of the distributions (*P* < 10^−12^, Kolmogorov-Smirnov test). Due to the uncertainties in electrophysiological recordings of very weak connections (below 0.1–0.25 mV in the neocortex), there is still controversy regarding the shape of the uPSP amplitude distribution in this region. Does the distribution smoothly approach zero with decreasing uPSP amplitude as suggested by the log-normal fit performed in Song et al. (2005) and predicted based on the model of multiplicative spine size dynamics (Loewenstein et al., 2011), does it increase as implied by the exponential distributions of spine head volumes (Mishchenko et al., 2010; Stepanyants and Escobar, 2011) and predicted by the unconstrained and *l*_1_ norm constrained models, or does it have a gap near zero as predicted by the *l*_0_ model? New, more sensitive experimental measurements are required to provide a definitive answer to these questions.

Both models of steady-state learning described in this study predict Gaussian decay of connection weight distributions in the region of strong connections. Contrary to this, distributions of uEPSP amplitudes in Figure 5 exhibit much heavier tails in the region beyond 1 mV. Several explanations have been previously proposed in order to account for this feature theoretically. Neurons may be operating below their critical capacity, or individual inputs to a neuron may be non-linearly transformed in the dendrites before they are summed in the cell body (Brunel et al., 2004). Heavy distribution tails have also been attributed to the fact that the number of potential presynaptic connections received by cortical neurons, though large [*N* ~ 5,000–10,000 (Lefort et al., 2009; Meyer et al., 2010)], is finite, while the theoretical results of this study were obtained in the *N* → ∞ limit. In fact, numerical simulations performed for *N* = 500 potential inputs can lead to heavier tails of connection weight distributions, supporting this interpretation (e.g., Figure 6D in Chapeton et al., 2012).

It was previously shown (see Figure 5C in Chapeton et al., 2012) that the unconstrained model provides a good fit to the IPSP distribution reported in Holmgren et al. (2003). Unfortunately, due to generally low counts in published PSP data for inhibitory neurons we had to refrain from examining such connections in this study. With future advances in optical methods such as glutamate uncaging (Packer and Yuste, 2011) and optogenetic tagging of genetically defined interneurons (Kvitsiani et al., 2013), it should be possible to study connections between multiple inhibitory and excitatory classes. Here, significant deviations in connection probabilities and shapes of PSP distributions from what is predicted by the theory could reveal connection types that are not directly involved in associative learning.

Our results show how neural networks may benefit from the presence of a small fraction of inhibitory neurons and connections. Figure 2 illustrates that a small fraction of inhibitory connections increases the capacity of the neurons for robust associative memory storage. This increase, however, comes at the expense of the overall connection weight in the *l*_0_ model or total number of functional connections in the *l*_1_ model, quantities that are likely to be directly related to the metabolic cost of the brain. It would be interesting to find out how different cortical areas balance network performance, measured in terms of information flow or memory storage capacity, with the metabolic cost associated with neuron firing, and number and weight of functional synaptic connections.

In this study, it was assumed that memory recall is a dynamic event in which activity of the network steps through a chain of predefined states. It is well known that in binary neural networks, such as the ones considered here, a chain of network states will inevitably terminate at an attractor in the form of a fixed point or a limit cycle. Hence, memory recall is bound to lead to a frozen network state, or states of cycling activity. With no external input, a network that is robust to small fluctuations could remain in the attractor state over a prolonged period of time, which is unrealistic. Thus, we think that external input, delivered via inter-areal, inter-hemispheric, and subcortical projections, must be responsible for reinitializing the network activity and initiating the recall of new associative memories.

The theoretical model presented in this study describes the effects of associative learning on synaptic connectivity in a steady-state of maximum memory storage capacity. We would like to emphasize that network connectivity in such a state need not be static. As the network is continually learning new associative memories (while forgetting some of the old ones) individual synaptic connections may potentiate or depress, new synaptic connections may be created, and the existing connections may be eliminated. Yet, the average features of steady-state connectivity must remain constant. What is more, these features are independent of the path taken by the network to reach the steady-state, making the problem theoretically tractable. Clearly, not all changes in synaptic connectivity can be described within the steady-state framework presented here. Changes which occur during development (Van Ooyen, 2011), follow injury or lesion (Butz and Van Ooyen, 2013), or accompany learning of new skills may perturb the network from the steady-state for prolonged periods of time (Ruediger et al., 2011). It is more difficult to model these non-equilibrium processes, as they require detailed knowledge of animal's experience and the learning rules involved.

### Conflict of interest statement

The authors declare that the research was conducted in the absence of any commercial or financial relationships that could be construed as a potential conflict of interest.

## Supplementary material

Text S1 **Detailed analytical and numerical solutions of the homeostatically constrained model of associative memory storage**.

Figure S1 **Validation of theoretical results with numerical simulations**.

Theoretical_Results.m. **Custom MatLab code for solving the unconstrained and *l*_0, 1_ constrained models**.
