# Supplementary material for: Effects of homeostatic constraints on associative memory storage and synaptic connectivity of cortical circuits
Source: Front Comput Neurosci. 2015 Jun 18;9:74. doi: 10.3389/fncom.2015.00074 (PMC4471370; doi:10.3389/fncom.2015.00074)
Supplement: Text S1 — Detailed analytical and numerical solutions of the homeostatically constrained model of associative memory storage. [file DataSheet1.PDF]

SUPPLEMENTARY MATERIAL

Here we formulate and solve a model of associative memory storage which incorporates a number of biologically motivated features of synaptic connectivity. This model is related to the perceptron (Rosenblatt, 1957;Minsky and Papert, 1988), but includes various generalizations and constraints. Specifically, the postsynaptic neuron can receive inputs from multiple excitatory or inhibitory cell classes which have distinct firing rates. The postsynaptic neuron robustly learns associations by modifying the input weights, while its firing threshold remains fixed. Finally, we consider two types of constraints on the input connection weights, l_0 norm constraint (fixed number of non-zero weight inputs), and l_1 norm constraint (fixed overall magnitude of input weights). The unconstrained case was previously solved by Chapeton et al. (Chapeton et al., 2012), and the results are included in the main text for comparison. Other models, which include only some of the constraints listed above, have been considered in a number of studies [see e.g. (Cover, 1965;Edwards and Anderson, 1975;Sherrington and Kirkpatrick, 1975;Gardner, 1988;Gardner and Derrida, 1988;Amit et al., 1989;Viswanathan, 1993;Brunel et al., 2004)].

The model

Consider a postsynaptic neuron receiving N potential inputs belonging to distinct classes (see Figure 1 of the main text). The inputs are classified based on their excitatory/inhibitory nature and their firing probabilities. The postsynaptic neuron must learn a set of m input-output associations, given the following:

1. The weights of input connections, J_j (j = 1,…, N), are sign-constrained as defined by their class, $J_j g_j \geq 0$, where g_j is +1 for excitatory inputs and -1 for inhibitory.
2. The neuron is constrained to have a fixed number of non-zero weight connections (l_0 norm constraint) or a fixed overall magnitude of connection weights (l_1 norm constraint).
3. The inputs, X_j^μ (μ = 1,…, m), and outputs, y^μ, are binary (0 or 1) and are randomly drawn from input-class dependent probability distributions.
4. The firing threshold, h, of the postsynaptic neuron is fixed and does not change during learning.
5. The associations, $\left\{X_j^\mu, y^\mu\right\}$, must be learned robustly, which is enforced through the robustness parameter, κ.

The model can be summarized mathematically as:

$$
\begin{aligned}
&\left(2y^{\mu}-1\right)\left(\sum_{j=1}^{N} J_j X_j^{\mu} - h\right) > \kappa \geq 0, \quad \mu = 1,\ldots,m \\
&\sum_{j=1}^{N} \left|J_j\right|^{l} = N w_l, \quad l = 0,1 \\
&J_j g_j \geq 0, \quad j = 1,\ldots,N \\
&\mathrm{Prob}\left(X_j^{\mu}\right) = \begin{cases} 1-f_j, & X_j^{\mu}=0 \\ f_j, & X_j^{\mu}=1 \end{cases}; \quad \mathrm{Prob}\left(y^{\mu}\right) = \begin{cases} 1-f_{out}, & y^{\mu}=0 \\ f_{out}, & y^{\mu}=1 \end{cases}
\end{aligned} \tag{1}
$$

In these expressions, f_j is the firing probability of input class j, f_{out} is the firing probability of the postsynaptic neuron, and l = 0 in the l_0 norm is defined in the limit of $l \rightarrow 0$.

In the large N limit, and under the assumption that the firing probabilities f_j and f_{out} do not scale with N, it can be shown that the average input to the postsynaptic neuron is of order h, and that the fluctuations about this average are of order κ (Chapeton et al., 2012). This fact motivates the following scaling of the model variables:

$$
J_j = \frac{h}{N}\tilde{J}_j; \quad \kappa = \frac{h}{\sqrt{N}}\tilde{\kappa}; \quad w_l = \tilde{w}_l \left(\frac{h}{N}\right)^{l} \tag{2}
$$

Here, the rescaled weights, $\tilde{J}_j$, robustness, $\tilde{\kappa}$, and parameter $\tilde{w}_l$ do not scale with N.

Substituting the rescaled variables into Eqs. (1) we arrive at expressions which are governed by only intensive parameters f_j, f_{out}, $\tilde{\kappa}$, and $\tilde{w}_l$:

$$
\begin{aligned}
&\left(2y^{\mu}-1\right)\left(\frac{1}{N}\sum_{j=1}^{N} \tilde{J}_j X_j^{\mu} - 1\right) > \frac{\tilde{\kappa}}{\sqrt{N}}, \quad \mu = 1,\ldots,m \\
&\frac{1}{N}\sum_{j=1}^{N} \left|\tilde{J}_j\right|^{l} = \tilde{w}_l, \quad l = 0,1 \\
&\tilde{J}_j g_j \geq 0, \quad j = 1,\ldots,N \\
&\mathrm{Prob}\left(X_j^{\mu}\right) = \begin{cases} 1-f_j, & X_j^{\mu}=0 \\ f_j, & X_j^{\mu}=1 \end{cases}; \quad \mathrm{Prob}\left(y^{\mu}\right) = \begin{cases} 1-f_{out}, & y^{\mu}=0 \\ f_{out}, & y^{\mu}=1 \end{cases}
\end{aligned} \tag{3}
$$

Solution of the model

In this section we outline the main steps for solving the model for the l_0 and l_1 cases. A more detailed calculation, which follows similar derivation steps, was previously published for the unconstrained case (Chapeton et al., 2012).

We begin by calculating the fraction of the connection weight space, $\Omega\left(X_j^\mu, y^\mu\right)$, in which Eqs. (3) hold for a given set of associations, $\left\{X_j^\mu, y^\mu\right\}$:

$$\Omega\left(X_j^\mu, y^\mu\right) = \frac{\int \prod_{j=1}^{N} d\tilde{J}_j \prod_{\mu=1}^{m} \theta\left(\left(2y^\mu - 1\right)\left(\frac{1}{N}\sum_{j=1}^{N}\tilde{J}_j X_j^\mu - 1\right) - \frac{\tilde{\kappa}}{\sqrt{N}}\right) \prod_{j=1}^{N}\theta\left(\tilde{J}_j g_j\right)\delta\left(\frac{1}{N}\sum_{j=1}^{N}\left|\tilde{J}_j\right|^l - \tilde{w}_l\right)}{\int \prod_{j=1}^{N} d\tilde{J}_j \delta\left(\frac{1}{N}\sum_{j=1}^{N}\left|\tilde{J}_j\right|^l - \tilde{w}_l\right)} \quad (4)$$

Here, θ denotes the Heaviside step function. The denominator in Eq. (4) can be calculated for arbitrary values of $l > 0$,

$$S_l = \int \prod_{j=1}^{N} d\tilde{J}_j \delta\left(\frac{1}{N}\sum_{j=1}^{N}\left|\tilde{J}_j\right|^l - \tilde{w}_l\right) = \frac{\left(\frac{2}{l}\Gamma\left(\frac{1}{l}\right)\left(\tilde{w}_l N\right)^{\frac{1}{l}}\right)^N}{\tilde{w}_l \Gamma\left(\frac{N}{l}\right)} \quad (5)$$

The typical fraction of the solution space volume, $\Omega_{typical}$, is defined through the averaging of $\ln\left(\Omega\left(X_j^\mu, y^\mu\right)\right)$ over the set of associations and is calculated by introducing n replica systems,

$$\ln\left(\Omega_{typical}\right) = \left\langle \ln\left(\Omega\left(X_j^\mu, y^\mu\right)\right)\right\rangle_{X_j^\mu, y^\mu} = \lim_{n\to 0}\frac{\left\langle \Omega\left(X_j^\mu, y^\mu\right)^n\right\rangle_{X_j^\mu, y^\mu} - 1}{n} \quad (6)$$

The quantity $\left\langle \Omega\left(X_j^\mu, y^\mu\right)^n\right\rangle_{X_j^\mu, y^\mu}$ in this expression is expressed in terms of a single multidimensional integral:

$$\left\langle \Omega\left(X_j^\mu, y^\mu\right)^n\right\rangle_{X_j^\mu, y^\mu} = S_l^{-n}\left\langle \int \prod_{a,j=1}^{n,N} d\tilde{J}_j^a \prod_{\mu,a=1}^{m,n}\theta\left(\left(2y^\mu - 1\right)\left(\frac{1}{N}\sum_{j=1}^{N}\tilde{J}_j^a X_j^\mu - 1\right) - \frac{\tilde{\kappa}}{\sqrt{N}}\right)\times \right.$$
$$\left. \prod_{j,a=1}^{N,n}\theta\left(\tilde{J}_j^a g_j\right)\prod_{a=1}^{n}\delta\left(\frac{1}{N}\sum_{j=1}^{N}\left|\tilde{J}_j^a\right|^l - \tilde{w}_l\right)\right\rangle_{X_j^\mu, y^\mu} \quad (7)$$

Below, we outline the main steps of calculation of Eq. (7). We begin by decoupling the averaging over the inputs and outputs, X_j^μ and y^μ, through the introduction of a new variable $\frac{\lambda^{a,\mu}}{\sqrt{N}} = \frac{1}{N}\sum_{j=1}^{N}\tilde{J}_j^a X_j^\mu - 1$:

$$\left\langle \Omega\left(X_j^{\mu}, y^{\mu}\right)^n \right\rangle_{X_j^{\mu}, y^{\mu}} = S_l^{-n} \int \prod_{a,j=1}^{n,N} d\tilde{J}_j^a \prod_{\mu,a=1}^{m,n} \frac{d\lambda^{a,\mu}}{\sqrt{N}} \left\langle \prod_{\mu,a=1}^{m,n} \theta\left(\left(2y^{\mu}-1\right)\lambda^{a,\mu} - \tilde{\kappa}\right) \right\rangle_{y^{\mu}} \times$$
$$\left\langle \prod_{\mu,a=1}^{m,n} \delta\left(1 + \frac{\lambda^{a,\mu}}{\sqrt{N}} - \frac{1}{N}\sum_{j=1}^{N} \tilde{J}_j^a X_j^{\mu}\right) \right\rangle_{X_j^{\mu}} \prod_{j,a=1}^{N,n} \theta\left(\tilde{J}_j^a g_j\right) \prod_{a=1}^{n} \delta\left(\frac{1}{N}\sum_{j=1}^{N} \left|\tilde{J}_j^a\right|^l - \tilde{w}_l\right) \tag{8}$$

Next, the step functions and the δ-functions are replaced with their Fourier representations,

$$\theta\left(\left(2y^{\mu}-1\right)\lambda^{a,\mu} - \tilde{\kappa}\right) = \int \frac{d'u^{a,\mu} d\hat{u}^{a,\mu}}{2\pi} e^{i\hat{u}^{a,\mu}\left(\left(2y^{\mu}-1\right)\lambda^{a,\mu} - \tilde{\kappa} - u^{a,\mu}\right)} \tag{9}$$

Symbol d' in this expression and thereafter is designated for 0 to ∞ integration, whereas d is used for integration from $-\infty$ to ∞.

After performing the averaging over the associations we arrive at:

$$\left\langle \Omega\left(X_j^{\mu}, y^{\mu}\right)^n \right\rangle_{X_j^{\mu}, y^{\mu}} = S_l^{-n} \int \prod_{\mu,a=1}^{m,n} \frac{d\lambda^{a,\mu} d\hat{\lambda}^{a,\mu}}{2\pi} \prod_{\mu,a=1}^{m,n} \frac{d'u^{a,\mu} d\hat{u}^{a,\mu}}{2\pi} \prod_{a=1}^{n} \frac{d\hat{k}^a}{2\pi / N} \times$$
$$e^{i\sum_{\mu,a=1}^{m,n}\left(\hat{\lambda}^{a,\mu}\left(\sqrt{N}+\lambda^{a,\mu}\right) - \hat{u}^{a,\mu}\left(u^{a,\mu}+\tilde{\kappa}\right)\right) - i\tilde{w}_l N \sum_{a=1}^{n}\hat{k}^a} \prod_{\mu=1}^{m}\left(f_{out} e^{i\sum_{a=1}^{n}\hat{u}^{a,\mu}\lambda^{a,\mu}} + (1-f_{out}) e^{-i\sum_{a=1}^{n}\hat{u}^{a,\mu}\lambda^{a,\mu}} \right) \times \tag{10}$$
$$\prod_{j=1}^{N}\left[\prod_{a=1}^{n} d\tilde{J}_j^a \prod_{\mu=1}^{m} e^{-if_j \frac{1}{\sqrt{N}}\sum_{a=1}^{n}\hat{\lambda}^{a,\mu}\tilde{J}_j^a + \frac{f_j^2 - f_j}{2}\left(\frac{1}{\sqrt{N}}\sum_{a=1}^{n}\hat{\lambda}^{a,\mu}\tilde{J}_j^a\right)^2} \prod_{a=1}^{n}\theta\left(\tilde{J}_j^a g_j\right) \prod_{a=1}^{n} e^{i\hat{k}^a \left|\tilde{J}_j^a\right|^l} \right]$$

Variables $\hat{\lambda}$ and $\hat{k}$ in this expression arise from Fourier representations of the two sets of δ-functions in Eq. (8).

At this point we introduce two sets of order parameters which make it possible to decouple the products containing indices j and μ,

$$\frac{1}{N}\sum_{j=1}^{N} f_j \tilde{J}_j^a = 1 + \frac{s^a}{\sqrt{N}}, \quad \frac{1}{N}\sum_{j=1}^{N} f_j\left(1-f_j\right)\tilde{J}_j^a \tilde{J}_j^b = q^{a,b} \tag{11}$$

The resulting integrals are calculated with the steepest descent method, assuming the existence of a replica symmetric saddle point, i.e. $\hat{k}^a = \hat{k}$, $s^a = s$, $\hat{s}^a = \hat{s}$, $q^{a,a} = q_0$, $q^{a\neq b} = q$, $\hat{q}^{a,a} = \hat{q}_0$, and $\hat{q}^{a\neq b} = \hat{q}$. Based on the insight from previous calculations (Chapeton et al., 2012), we introduce

the following substitutions, $t=-i\hat{q}$, $y=\sqrt{f_j\left(1-f_j\right)t}\tilde{J}$, $u_\pm=\frac{\tilde{\kappa}\pm s}{\sqrt{2q}}$, $\varepsilon=\frac{q_0-q}{q}$, $z=\frac{i\hat{s}}{2\sqrt{t}}$, $\delta=\frac{t+i\hat{q}_0}{t}$, $\eta=-i\hat{k}t^{-l/2}$, and give a summary of the results:

$$
\begin{aligned}
&\frac{\ln\left(\Omega_{typical}\right)}{N}=-\ln\left(\frac{2}{l}\Gamma\left(\frac{1}{l}\right)\left(l\tilde{w}_l e\right)^{\frac{1}{l}}\right)-\tilde{w}_l\eta t^{\frac{l}{2}}+2z\sqrt{t}+\frac{2\tilde{\kappa}^2}{\left(u_+ +u_-\right)^2}\left(\delta-\varepsilon+\varepsilon\delta\right)t+\\
&\alpha G_E(\varepsilon,u_-,u_+)+G_S(z,\delta,\eta,t)\\
&G_E(\varepsilon,u_-,u_+)=f_{out}\int\frac{dx}{\sqrt{\pi}}e^{-x^2}\ln\left(\frac{1}{2}\left(1+\operatorname{erf}\left(\frac{x-u_-}{\sqrt{\varepsilon}}\right)\right)\right)+(1-f_{out})\int\frac{dx}{\sqrt{\pi}}e^{-x^2}\ln\left(\frac{1}{2}\left(1+\operatorname{erf}\left(\frac{x-u_+}{\sqrt{\varepsilon}}\right)\right)\right)\\
&G_S\left(z,\delta,\eta,t\right)=\frac{1}{N}\sum_{j=1}^{N}\left(\int\frac{dx}{\sqrt{\pi}}e^{-x^2}\ln\left(\int d'y e^{-\delta y^2+\left(f_j\left(1-f_j\right)\right)^{-\frac{l}{2}}\eta y^l+2\left(x-\frac{f_j g_j}{\sqrt{f_j\left(1-f_j\right)}}z\right)y}\right)-\ln\sqrt{f_j\left(1-f_j\right)t}\right)
\end{aligned}
$$

(12)

In this expression $\alpha=m/N$ is referred to as the associative memory storage capacity, while the variables $\{\varepsilon,u_-,u_+,z,\delta,\eta,t\}$ are defined through the following seven saddle-point equations:

$$
\begin{aligned}
&-\frac{\partial G_E(\varepsilon,u_-,u_+)}{\partial u_-}+\frac{\partial G_E(\varepsilon,u_-,u_+)}{\partial u_+}=0\\
&\frac{\partial G_E(\varepsilon,u_-,u_+)}{\partial\varepsilon}=\frac{2\tilde{\kappa}^2}{\alpha\left(u_+ +u_-\right)^2}t\left(1-\delta\right)\\
&\frac{\partial G_E(\varepsilon,u_-,u_+)}{\partial\varepsilon}\left(1+\varepsilon\right)+\frac{1}{2}\frac{\partial G_E(\varepsilon,u_-,u_+)}{\partial u_-}u_-+\frac{1}{2}\frac{\partial G_E(\varepsilon,u_-,u_+)}{\partial u_+}u_+=\frac{2\tilde{\kappa}^2}{\alpha\left(u_+ +u_-\right)^2}t\\
&\frac{\partial G_S\left(z,\delta,\eta,t\right)}{\partial\eta}t^{-\frac{l}{2}}=\tilde{w}_l\\
&\frac{\partial G_S(z,\delta,\eta,t)}{\partial z}=-2\sqrt{t}\\
&\frac{\partial G_S(z,\delta,\eta,t)}{\partial\delta}=-\frac{2\tilde{\kappa}^2}{\left(u_+ +u_-\right)^2}\left(1+\varepsilon\right)t\\
&-\frac{\partial G_S(z,\delta,\eta,t)}{\partial z}\frac{z}{2}+\frac{\partial G_S(z,\delta,\eta,t)}{\partial\delta}\left(1-\delta\right)-\frac{\partial G_S(z,\delta,\eta,t)}{\partial\eta}\frac{l}{2}\eta+\frac{\partial G_S(z,\delta,\eta,t)}{\partial t}t=-\frac{2\tilde{\kappa}^2}{\left(u_+ +u_-\right)^2}t
\end{aligned}
$$

(13)

Critical capacity

At the critical associative memory storage capacity, the saddle point Eqs. (13) can be simplified because $\Omega_{typical}$ tends to zero, and therefore, $\left(q_0 - q\right)$ goes to zero as well. In this limit the seven saddle point equations can be expanded asymptotically to the leading orders in $1/\left(q_0 - q\right)$. After eliminating *t*, *δ*, and *η* from these equations, and introducing a new variable *x*, which is related to *α* through Eq. (17), we arrive at the final result:

$$
\begin{aligned}
&l = 0,1 \text{ norm constrained cases:}\\
&f_{out} I_1\left(-u_-, 0\right) = (1 - f_{out}) I_1\left(-u_+, 0\right)\\
&\frac{1}{N}\sum_{i=1}^{N}\left(\frac{1}{\sqrt{f_i(1-f_i)}}\right)^l I_l\left(g_i z\sqrt{\frac{f_i}{1-f_i}} + \frac{\left(\tilde{\kappa}x - z\right)}{\tilde{w}_l\sqrt{f_i(1-f_i)}}\delta_{l,1}, \sqrt{\frac{2\left(\tilde{\kappa}x - z\right)Q}{\tilde{w}_l}}\delta_{l,0}\right) = \tilde{w}_l Q^l\\
&\frac{1}{N}\sum_{i=1}^{N}\frac{f_i g_i}{\sqrt{f_i(1-f_i)}} I_1\left(g_i z\sqrt{\frac{f_i}{1-f_i}} + \frac{\left(\tilde{\kappa}x - z\right)}{\tilde{w}_l\sqrt{f_i(1-f_i)}}\delta_{l,1}, \sqrt{\frac{2\left(\tilde{\kappa}x - z\right)Q}{\tilde{w}_l}}\delta_{l,0}\right) = Q\\
&\frac{1}{N}\sum_{i=1}^{N} I_2\left(g_i z\sqrt{\frac{f_i}{1-f_i}} + \frac{\left(\tilde{\kappa}x - z\right)}{\tilde{w}_l\sqrt{f_i(1-f_i)}}\delta_{l,1}, \sqrt{\frac{2\left(\tilde{\kappa}x - z\right)Q}{\tilde{w}_l}}\delta_{l,0}\right) = \frac{2Q^2\tilde{\kappa}^2}{\left(u_+ + u_-\right)^2}\\
&Q = 2\frac{\left(u_+ + u_-\right)}{\tilde{\kappa}}\frac{f_{out} I_0\left(-u_-, 0\right) + (1 - f_{out}) I_0\left(-u_+, 0\right)}{f_{out} I_1\left(-u_-, 0\right) + (1 - f_{out}) I_1\left(-u_+, 0\right)} x\\
&u_+ + u_- \geq 0; \quad x \geq 0; \quad \left(\tilde{\kappa}x - z\right)\delta_{l,0} \geq 0
\end{aligned}
\qquad (14)
$$

In comparison, the corresponding equations for the unconstrained case, written in the notation of Eq. (14), have the form (Chapeton et al., 2012):

$$
\begin{aligned}
&\text{Unconstrained case:}\\
&f_{out} I_1\left(-u_-, 0\right) = (1 - f_{out}) I_1\left(-u_+, 0\right)\\
&z = \tilde{\kappa}x\\
&\frac{1}{N}\sum_{j=1}^{N}\frac{f_j g_j}{\sqrt{f_j\left(1-f_j\right)}} I_1\left(\frac{f_j g_j z}{\sqrt{f_j\left(1-f_j\right)}}, 0\right) = Q\\
&\frac{1}{N}\sum_{j=1}^{N} I_2\left(\frac{f_j g_j z}{\sqrt{f_j\left(1-f_j\right)}}, 0\right) = \frac{2Q^2\tilde{\kappa}^2}{\left(u_+ + u_-\right)^2}\\
&u_+ + u_- \geq 0; \quad x \geq 0
\end{aligned}
\qquad (15)
$$

The following special functions were used in Eqs. (14) and (15):

$$
\begin{aligned}
I_n(a,b) &= \int_0^\infty \frac{dy}{\sqrt{\pi}} e^{-(y+b+a)^2} \left(y+b+\sqrt{(y+b)^2-b^2} \right)^n \\
I_0(a,b) &= \frac{1}{2}\left(1-\operatorname{erf}(a+b)\right) \\
I_1(a,0) &= \frac{1}{\sqrt{\pi}} e^{-a^2} - a\left(1-\operatorname{erf}(a)\right) \\
I_2(a,0) &= -\frac{2a}{\sqrt{\pi}} e^{-a^2} + \left(2a^2+1\right)\left(1-\operatorname{erf}(a)\right)
\end{aligned}
\tag{16}
$$

The solution of Eqs. (14) defines the critical capacity in terms of the model parameters,

$$
\alpha_c\left(\{g_j\},\{f_j\}, f_{out}, \tilde{\kappa}, \tilde{w}_l\right) = 2x^2 \frac{\left(f_{out} I_2(-u_-,0) + (1-f_{out}) I_2(-u_+,0)\right)}{\left(f_{out} I_1(-u_-,0) + (1-f_{out}) I_1(-u_+,0)\right)^2}. \tag{17}
$$

Eqs. (14-17) were solved with custom MatLab code (Theoretical_Results.m of Supporting Materials) to produce the results of the main text.

Distribution of input weights at critical capacity

The probability density function for input weights can be written in general form as:

$$
p_i\left(\tilde{J}\right) = \left\langle \frac{\int \prod_{j=1}^{N} d\tilde{J}_j \delta\left(\tilde{J}_i - \tilde{J}\right) \prod_{\mu=1}^{m} \theta\left(\left(2y^\mu - 1\right)\left(\frac{1}{N}\sum_{j=1}^{N} \tilde{J}_j X_j^\mu - 1 \right) - \frac{\tilde{\kappa}}{\sqrt{N}} \right) \prod_{j=1}^{N} \theta\left(\tilde{J}_j g_j\right) \delta\left(\frac{1}{N}\sum_{j=1}^{N} \left|\tilde{J}_j\right|^l - \tilde{w}_l \right)}{\Omega\left(X_j^\mu, y^\mu\right) \int \prod_{j=1}^{N} d\tilde{J}_j \delta\left(\frac{1}{N}\sum_{j=1}^{N} \left|\tilde{J}_j\right|^l - \tilde{w}_l \right)} \right\rangle_{X_j^\mu, y^\mu} \tag{18}
$$

Eq. (18) can be cast in a form that closely resembles Eq. (7), allowing one to use the results from the previous sections. To this end, we introduce *n* replicas and take the limit of $n \to 0$ after averaging over the associations,

$$
\begin{aligned}
p_i\left(\tilde{J}\right) = \lim_{n\to 0} \Bigg\langle \int \prod_{a,j=1}^{n,N} d\tilde{J}_j^a \delta\left(\tilde{J}_i^{a=1} - \tilde{J}\right) \prod_{\mu,a=1}^{m,n} \theta\left(\left(2y^\mu - 1\right)\left(\frac{1}{N}\sum_{j=1}^{N} \tilde{J}_j^a X_j^\mu - 1 \right) - \frac{\tilde{\kappa}}{\sqrt{N}} \right) \times \\
\prod_{j,a=1}^{N,n} \theta\left(\tilde{J}_j^a g_j\right) \prod_{a=1}^{n} \delta\left(\frac{1}{N}\sum_{j=1}^{N} \left|\tilde{J}_j^a\right|^l - \tilde{w}_l \right) \Bigg\rangle_{X_j^\mu, y^\mu}
\end{aligned}
\tag{19}
$$

Following the steps described in Eqs. (7-12) we arrive at:

$$p_i\left(\tilde{J}\right)=\frac{1}{\sqrt{\pi}}\int_{-\infty}^{\infty}dxe^{-x^2}\frac{e^{\eta\left|\tilde{J}\sqrt{t}\right|^l+2\sqrt{t}\left(x\sqrt{f_i(1-f_i)}-f_i g_i z\right)g_i\tilde{J}-f_i(1-f_i)\delta t\tilde{J}^2}}{\int d'\tilde{J}_i^1 e^{\eta\left(\tilde{J}_i^1\sqrt{t}\right)^l+2\sqrt{t}\left(x\sqrt{f_i(1-f_i)}-f_i g_i z\right)\tilde{J}_i^1-f_i(1-f_i)\delta t\left(\tilde{J}_i^1\right)^2}} \tag{20}$$

At critical capacity, Eq. (20) can be simplified, leading to the probability density functions of connection weights for the various input types. A characteristic feature of these distributions is that they contain finite fractions of zero-weight connections:

$l=0,1$ norm constrained cases:

$$\begin{aligned}
&p_i\left(\tilde{J}\right)=\theta\left(g_i\tilde{J}\right)\frac{1}{\sqrt{2\pi}\Sigma_i}\left(1-\frac{\left(\tilde{\kappa}x-z\right)Q}{\tilde{w}_l}\frac{\Sigma_i^2}{\tilde{J}^2}\delta_{l,0}\right)_+ e^{-\left(\frac{\tilde{J}}{\sqrt{2}\Sigma_i}+z\sqrt{\frac{f_i}{1-f_i}}+\frac{\left(\tilde{\kappa}x-z\right)}{\tilde{w}_l\sqrt{f_i(1-f_i)}}\left(g_i\delta_{l,1}+\frac{\delta_{l,0}}{\tilde{J}}\right)\right)^2}+\\
&I_0\left(-g_i z\sqrt{\frac{f_i}{1-f_i}}-\frac{\left(\tilde{\kappa}x-z\right)}{\tilde{w}_l\sqrt{f_i\left(1-f_i\right)}}\delta_{l,1}-\sqrt{\frac{2\left(\tilde{\kappa}x-z\right)Q}{\tilde{w}_l}}\delta_{l,0},0\right)\delta\left(\tilde{J}\right)\\
&\Sigma_i=\frac{\sqrt{2}}{\sqrt{f_i(1-f_i)Q}}
\end{aligned} \tag{21}$$

Parameters Σ_i in this expression describe the widths of the input weight distributions. Plus sign in the subscript of the first equation in (21) denotes the positive part function. Note that in the l_0 case this function leads to gaps in the connection weight distributions. In this case, the postsynaptic neuron at critical capacity does not have weak connections, i.e. connection weights smaller than $\Sigma_i\sqrt{\left(\tilde{\kappa}x-z\right)Q/\tilde{w}_0}$ in magnitude. We would like to mention that for a single class of sign-unconstrained but l_0 norm constrained inputs, a gap in the distribution of connection weights was previously reported by Bouten et al. (Bouten et al., 1990).

The corresponding probability density functions in the unconstrained case, written in the notation of Eq. (21), have the form (Chapeton et al., 2012):

Unconstrained case:

$$p_i\left(\tilde{J}\right)=\theta\left(g_i\tilde{J}\right)\frac{1}{\sqrt{2\pi}\Sigma_i}e^{-\left(\frac{\tilde{J}}{\sqrt{2}\Sigma_i}+z\sqrt{\frac{f_i}{1-f_i}}\right)^2}+I_0\left(-g_i z\sqrt{\frac{f_i}{1-f_i}},0\right)\delta\left(\tilde{J}\right) \tag{22}$$

Connection probabilities and distributions of non-zero connection weights

Connection probabilities and uPSP amplitudes are routinely measured in electrophysiological experiments. Connection probabilities in the model, P^{con}, are defined as the fractions of non-zero weight inputs. These fractions can be obtained from Eqs. (21) and (22):

$l = 0,1$ norm constrained cases:

$$P_i^{con} = I_0\left(g_i z\sqrt{\frac{f_i}{1-f_i}} + \frac{(\tilde{\kappa}x - z)}{\tilde{w}_l\sqrt{f_i(1-f_i)}}\delta_{l,1} + \sqrt{\frac{2(\tilde{\kappa}x-z)Q}{\tilde{w}_l}}\delta_{l,0}, 0\right)$$

Unconstrained case: (23)

$$P_i^{con} = I_0\left(g_i z\sqrt{\frac{f_i}{1-f_i}}, 0\right)$$

Probability density functions of non-zero input weights in the model, which correspond to experimental distributions of uPSP amplitudes, are derived from Eqs. (21) and (22) as well:

$l = 0,1$ norm constrained cases:

$$p_i(\tilde{J}) = \frac{\left(1 - \frac{(\tilde{\kappa}x-z)Q}{\tilde{w}_l}\frac{\Sigma_i^2}{\tilde{J}^2}\delta_{l,0}\right)_+ e^{-\left(\frac{\tilde{J}}{\sqrt{2}\Sigma_i} + z\sqrt{\frac{f_i}{1-f_i}} + \frac{(\tilde{\kappa}x-z)}{\tilde{w}_l\sqrt{f_i(1-f_i)}}\left(g_i\delta_{l,1} + \frac{\delta_{l,0}}{\tilde{J}}\right)\right)^2}}{\sqrt{2\pi}\Sigma_i I_0\left(g_i z\sqrt{\frac{f_i}{1-f_i}} + \frac{(\tilde{\kappa}x - z)}{\tilde{w}_l\sqrt{f_i(1-f_i)}}\delta_{l,1} + \sqrt{\frac{2(\tilde{\kappa}x-z)Q}{\tilde{w}_l}}\delta_{l,0}, 0\right)}$$

Unconstrained case: (24)

$$p_i(\tilde{J}) = \frac{e^{-\left(\frac{\tilde{J}}{\sqrt{2}\Sigma_i} + z\sqrt{\frac{f_i}{1-f_i}}\right)^2}}{\sqrt{2\pi}\Sigma_i I_0\left(g_i z\sqrt{\frac{f_i}{1-f_i}}, 0\right)}$$

We note that the connection weight distributions for the unconstrained and l_1 norm constrained cases are Gaussian functions truncated at zero. The l_0 norm distribution is significantly different; it is non-Gaussian and contains a gap near zero.

Numerical solutions with methods of convex optimization

Here we describe the numerical algorithms used to validate the solutions to the unconstrained and l_1 norm constrained problems, Eq. (3). These simulations were used to generate the results of Figure S1A, C, D, F. Both problems are convex, and hence, can be solved within the standard constrained optimization framework. To this end, we turn the inequalities of Eq. (3) into soft constraints to make the problem feasible, and add a small regularizing potential to make the solution unique:

$$
\begin{aligned}
&\min_{s,\tilde{J}_j}\left(s+\frac{\varepsilon}{N}\sum_{j=1}^{N}\tilde{J}_j^{\,2}\right); \quad \varepsilon\geq 0\\
&-\frac{\left(2y^{\mu}-1\right)}{N}\sum_{j=1}^{N}\tilde{J}_j X_j^{\mu}+\left(2y^{\mu}-1\right)+\frac{\tilde{\kappa}}{\sqrt{N}}\leq s, \quad \mu=1,\ldots,m\\
&-g_j\tilde{J}_j\leq s, \quad j=1,\ldots,N\\
&\frac{1}{N}\sum_{j=1}^{N}\left|\tilde{J}_j\right|-\tilde{w}_1=0, \quad l=1
\end{aligned}
\tag{25}
$$

This problem can be solved in the primal-dual Lagrangian framework (Boyd and Vandenberghe, 2004). We construct the primal Lagrange function by combining the objective function of Eq. (25) with the constraints weighted by Lagrange multipliers:

$$
\begin{aligned}
&L_p\left(s,\tilde{J}_j;\alpha_\mu,\beta_j,\gamma\right)=s+\frac{\varepsilon}{N}\sum_{j=1}^{N}\tilde{J}_j^{\,2}+\sum_{\mu=1}^{m}\alpha_\mu\left(-\frac{\left(2y^{\mu}-1\right)}{N}\sum_{j=1}^{N}\tilde{J}_j X_j^{\mu}+\left(2y^{\mu}-1\right)+\frac{\tilde{\kappa}}{\sqrt{N}}-s\right)-\\
&\sum_{j=1}^{N}\beta_j\left(g_j\tilde{J}_j+s\right)+\gamma\delta_{l,1}\left(\frac{1}{N}\sum_{j=1}^{N}\left|\tilde{J}_j\right|-\tilde{w}_1\right)\\
&\alpha_\mu\geq 0; \quad \beta_j\geq 0
\end{aligned}
\tag{26}
$$

The dual Lagrangian and the optimal values of connection weights are calculated as follows:

$$
\begin{aligned}
&L_d\left(\alpha_\mu,\beta_j,\gamma\right)=\min_{s,\tilde{J}_j}L_p\left(s,\tilde{J}_j;\alpha_\mu,\beta_j,\gamma\right)=\sum_{\mu=1}^{m}\alpha_\mu\left(\left(2y^{\mu}-1\right)+\frac{\tilde{\kappa}}{\sqrt{N}}\right)-\gamma\tilde{w}_1\delta_{l,1}-\\
&\frac{1}{4\varepsilon N}\sum_{j=1}^{N}\left(\sum_{\mu=1}^{m}\alpha_\mu\left(2y^{\mu}-1\right)X_j^{\mu}+\beta_j g_j-\gamma g_j\delta_{l,1}\right)^2+\begin{cases}0, & \sum_{\mu=1}^{m}\alpha_\mu+\sum_{j=1}^{N}\beta_j=1\\ -\infty, & else\end{cases}\\
&\tilde{J}_j=\frac{1}{2\varepsilon}\left(\sum_{\mu=1}^{m}\alpha_\mu\left(2y^{\mu}-1\right)X_j^{\mu}+\beta_j g_j-\gamma g_j\delta_{l,1}\right)
\end{aligned}
\tag{27}
$$

This leads to a relatively simple associated dual problem:

$$
\begin{aligned}
&\max_{\alpha_\mu,\beta_\mu,\gamma}L_d\left(\alpha_\mu,\beta_j,\gamma\right)\\
&\alpha_\mu\geq 0; \quad \beta_j\geq 0\\
&\alpha_\mu\left(-\frac{\left(2y^{\mu}-1\right)}{N}\sum_{j=1}^{N}\tilde{J}_j X_j^{\mu}+\left(2y^{\mu}-1\right)+\frac{\tilde{\kappa}}{\sqrt{N}}-s\right)=0\\
&\beta_j\left(g_j\tilde{J}_j+s\right)=0
\end{aligned}
\tag{28}
$$

Maximization over the Lagrange multiplier γ can be carried out explicitly,

$$
\begin{aligned}
&\max_{\alpha_\mu,\beta_j}\left(\sum_{\mu=1}^{m}\alpha_\mu\left(\left(2y^\mu-1\right)+\frac{\tilde{\kappa}}{\sqrt{N}}\right)-\frac{\tilde{w}_1\delta_{l,1}}{N}\sum_{j=1}^{N}A_j-\frac{1}{4\varepsilon N}\sum_{j=1}^{N}\left(A_j-\frac{\delta_{l,1}}{N}\sum_{j=1}^{N}A_j\right)^2\right)\\
&A_j=g_j\sum_{\mu=1}^{m}\alpha_\mu\left(2y^\mu-1\right)X_j^\mu+\beta_j\\
&\alpha_\mu\geq 0;\quad \beta_j\geq 0\\
&\alpha_\mu\left(-\frac{\left(2y^\mu-1\right)}{N}\sum_{j=1}^{N}\tilde{J}_jX_j^\mu+\left(2y^\mu-1\right)+\frac{\tilde{\kappa}}{\sqrt{N}}-s\right)=0\\
&\beta_j\left(g_j\tilde{J}_j+s\right)=0\\
&\sum_{\mu=1}^{m}\alpha_\mu+\sum_{j=1}^{N}\beta_j=1
\end{aligned}
\tag{29}
$$

The resulting optimization problem was solved by using a custom MatLab algorithm, generating the numerical results of Figure S1. The connection weights were determined based on the optimum values of the Lagrange multipliers α and β according to Eq. (27).

The critical capacity resulting from these simulations is in good agreement with the theoretical calculations (Figure S1A, C). Numerical simulations also reproduce the overall shapes of theoretical distributions of connection weights (Figure S1D, F). However, a small deviation in distribution width was observed in the unconstrained model. We attribute this discrepancy to the effect of a finite number of inputs (N_{inh} = 75 and N_{exc} = 425). Consistent with this interpretation, the deviation is larger for inhibitory inputs, while it is absent in the l_1 case.

Numerical solutions with perceptron-type learning rules

In addition to the above described theoretical solutions and numerical solutions based on convex optimization, the three models considered in this study were also solved numerically with modified perceptron learning rules (see Figure S1B, E and Figure 4 of the main text). This was necessary for two reasons. First, such numerical solutions establish biological plausibility of the steady-state learning hypothesis, which among other things assumes that neurons can reach the state of critical capacity. Second, since the l_0 norm constrained problem is non-convex, and hence, cannot be solved with convex optimization, we had to find an alternative numerical strategy for this case.

Such numerical simulations in the unconstrained case utilized perceptron learning rule (Engel and Broeck, 2001) with the addition of sign constraints on the weights of excitatory and inhibitory connections. This algorithm was previously described in (Chapeton et al., 2012). To incorporate the l_1 norm constraint, we uniformly rescaled connection weights after every perceptron learning step. In the l_0 norm constraint case, which is a constraint on the number of

non-zero weight connections, we monitored this number after every perceptron learning step. If at any point the number of non-zero weight connections exceeded the value allowed by the constraint, a single weakest connection (excitatory or inhibitory) was set to zero. These three modified perceptron learning rules can be summarized as follows:

$$\begin{aligned}
&\tilde{J}_k \mapsto \tilde{J}_k + \frac{\beta}{\sqrt{N}}\left(2y^{\mu} - 1\right) X_k^{\mu}, \quad k = 1,\ldots,N \\
&\tilde{J}_k \mapsto \tilde{J}_k \theta\left(\tilde{J}_k g_k\right) \\
&l = 0 \text{ case:} \quad \tilde{J}_{\arg\min_j\left(\left|\tilde{J}_j\right|\right)} \mapsto 0, \quad \text{if } \frac{1}{N}\sum_{j=1}^{N}\theta\left(\left|\tilde{J}_j\right|\right) > \tilde{w}_0 \\
&l = 1 \text{ case:} \quad \tilde{J}_k \mapsto \frac{\tilde{w}_1}{\frac{1}{N}\sum_{j=1}^{N}\left|\tilde{J}_j\right|}\tilde{J}_k
\end{aligned} \tag{30}$$

These expressions describe the update of input weights, $\tilde{J}_k$, in response to the presentation of a single association μ, which has not been learned by the neuron. Parameter β defines the learning rate. This parameter was set to 0.05 in all numerical simulations. Associations were selected randomly for learning, and for each association the above update rule was applied until the association was learned. Numerical algorithms in all three cases were allowed to continue until the solution satisfying all the constraints was found, or until the maximum number of iterations (10^6) was reached. There is no proof of convergence for these procedures, but the results underestimate the theoretical critical capacity by no more than 5%-15% (see Figure S1B).

Figure legends

Figure S1: Validation of theoretical results with numerical simulations. **A-C.** Critical capacity as a function of the fraction of potential inhibitory inputs, N_{inh}/N. Theoretical results (solid lines) are generally consistent with the results of numerical simulations performed for N = 500 potential inputs (solid dots). Results for various values of the robustness parameter κ are shown with different colors. Due to the difficulties associated with solving non-convex optimization problems, comprehensive numerical simulations in the l_0 case (B) were performed for two values of κ only. Numerous spot-checks were performed to validate the general trends of other theoretical curves in the l_0 case (results not shown). **D-F.** Distributions of excitatory and inhibitory connection weights for $N_{inh}/N = 0.15$, $\sqrt{N}\kappa/h = 0.5$, and $f_{inh,\,exc} = 0.5$. Values of the constraints are $w_0 = 0.2$ in E and $Nw_1/h = 3$ in F. The theoretical results are in green and the results of numerical simulations performed for N = 500 potential inputs are in blue. Small deviations of numerical distributions from theoretical are attributed to relatively small numbers of excitatory and inhibitory inputs used in simulations. Corresponding theoretical and numerical fractions of non-zero weight connections, P^{con}, are shown in green and blue respectively.

References

Amit, J.A., Campbell, C., and Wong, K.Y.M. (1989). The interaction space of neural networks with sign_constrained synapses. *J. Phys. A: Math. Gen.* 22**,** 4687-4693.

Bouten, M., Engel, A., Komoda, A., and Serneels, R. (1990). Quenched Versus Annealed Dilution in Neural Networks. *Journal of Physics a-Mathematical and General* 23**,** 4643-4657. doi: Doi 10.1088/0305-4470/23/20/025.

Boyd, S.P., and Vandenberghe, L. (2004). *Convex optimization.* Cambridge, UK ; New York: Cambridge University Press.

Brunel, N., Hakim, V., Isope, P., Nadal, J.P., and Barbour, B. (2004). Optimal information storage and the distribution of synaptic weights: perceptron versus Purkinje cell. *Neuron* 43**,** 745-757.

Chapeton, J., Fares, T., Lasota, D., and Stepanyants, A. (2012). Efficient associative memory storage in cortical circuits of inhibitory and excitatory neurons. *Proc Natl Acad Sci U S A* 109**,** E3614-3622. doi: 1211467109 [pii]
10.1073/pnas.1211467109.

Cover, T.M. (1965). Geometrical and statistical properties of systems of linear inequalities with applications in pattern recognition. *IEEE Trans. EC* 14**,** 326-334.

Edwards, S.F., and Anderson, P.W. (1975). Theory of spin glasses. *J. Phys. F: Metal Phys.* 5**,** 965-974.

Engel, A., and Broeck, C.V.D. (2001). *Statistical mechanics of learning.* Cambridge, UK ; New York, NY: Cambridge University Press.

Gardner, E. (1988). The space of interactions in neural network models. *J. Phys. A: Math. Gen.* 21**,** 257-270.

Gardner, E., and Derrida, B. (1988). Optimal storage properties of neural network models. *J. Phys. A: Math. Gen.* 21**,** 271-284.

Minsky, M.L., and Papert, S. (1988). *Perceptrons : an introduction to computational geometry.* Cambridge, Mass.: MIT Press.

Rosenblatt, M. (1957). The Multidimensional Prediction Problem. *Proc Natl Acad Sci U S A* 43**,** 989-992.

Sherrington, D., and Kirkpatrick, S. (1975). Solvable model of a spin glass. *Physical Review Letters* 35**,** 1792-1796.

Viswanathan, R.R. (1993). Sign-Constrained Synapses and Biased Patterns in Neural Networks. *Journal of Physics a-Mathematical and General* 26**,** 6195-6203.
