# Supplementary figures and images for: Effects of homeostatic constraints on associative memory storage and synaptic connectivity of cortical circuits
Source: Front Comput Neurosci. 2015 Jun 18;9:74. doi: 10.3389/fncom.2015.00074 (PMC4471370; doi:10.3389/fncom.2015.00074)

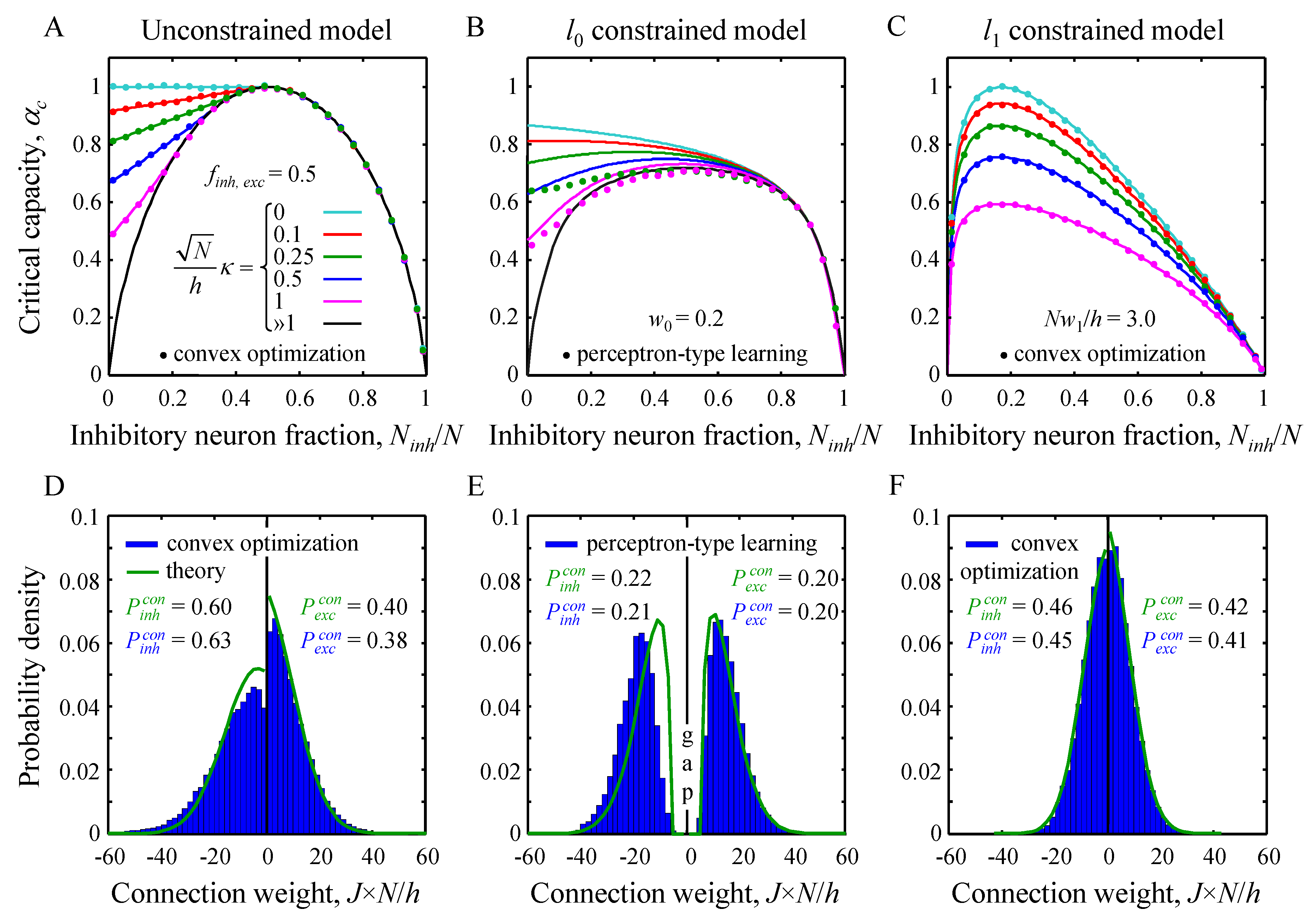

Supplement: Figure S1 — Validation of theoretical results with numerical simulations. [file Image1.TIFF]
